# Design and synthesis of thiahelicenes for molecular electronics

**DOI:** 10.3389/fchem.2024.1471413

**Published:** 2024-10-14

**Authors:** Bianca C. Baciu, Pawel J. Bronk, Albert Guijarro

**Affiliations:** Instituto Universitario de Síntesis Orgánica and Departamento de Química Orgánica, Campus de San Vicente del Raspeig, Universidad de Alicante, Alicante, Spain

**Keywords:** organic synthesis, dithiahelicene, photocyclization, bond topology, molecular solenoid, density functional theory binding energy, gold electrode

## Abstract

The conductance of a tunneling electron through a π-conjugated molecule may be affected by the presence of different pathways in the orbital structure of the molecule, resulting in the constructive or destructive interference of the molecular wave function. This quantum interference (QI) directly translates into enhancement or suppression of conductance and offers the possibility of controlling this phenomenon through tailored synthesis. Hence, we set up synthetic methodologies to access a series of thiophene-fused helicenes with a well-defined positioning of the sulfur atoms, which control the occurrence of conducting, linearly conjugated as well as disrupted, cross-conjugated pathways. We describe these synthetic strategies and relate the expected electronic transport through our molecules to three key variables: a) the *exo*-/*endo*-topology of the S atom within the ring; b) the parity (odd/even) of the overall number of rings conforming to the helicene; and c) the size of the circuit. This series ranks from [7] to [11] fused rings, having both *exo*-, *endo*-, or mixed *exo-endo-*topology. Comparison of homologous dithiahelicenes with size-tunable highest occupied molecular orbital (HOMO)/lowest unoccupied molecular orbital (LUMO) energies allows us to isolate the key variable of the bond topology from other electronic properties and face the study of QI in helically conjugated molecules. Understanding and tuning the conductance in such molecular solenoids is the main purpose of this work.

## Graphical Abstract

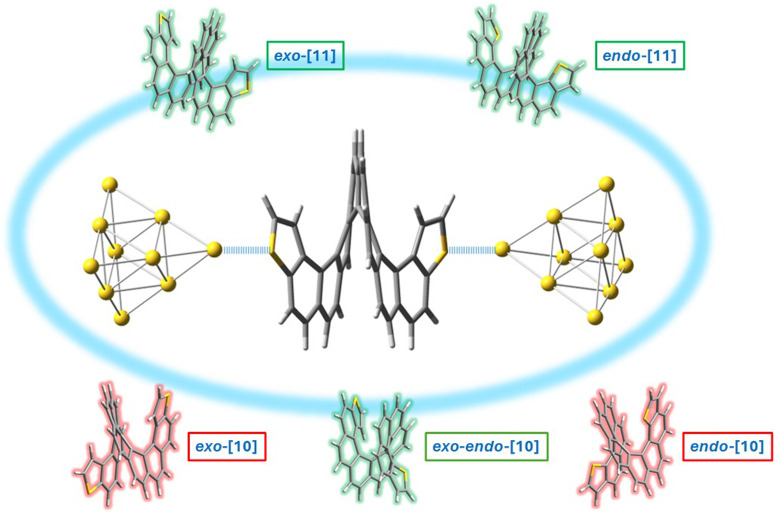

## 1 Introduction

For over a century, helicenes—non-planar, all-aromatic helical-shaped molecules—have emerged as captivating subjects for advanced research (Chen and Shen, 2017) (Crassous et al., 2022). Due to their twisted structures, these intriguing compounds exhibit unique properties that place them as promising candidates in different areas of scientific exploration (Sahasithiwat et al., 2010); (Hatakeyama et al., 2012); (Taniguchi et al., 2000); (Ernst et al., 2001); (Sanvito, 2011). This work is focused on their molecular electronics properties, aiming to build electronic devices using individual molecules as one of the ultimate goals in electronics. Molecular-scale electronics is a constantly growing research domain. It not only enhances investigations in this field through device miniaturization, resulting in unprecedented efficiencies, but also provides an ideal window for exploring the intrinsic properties of materials at the molecular level. Along the way, intriguing quantum effects are often harnessed. In the molecular electronic junction, the molecule is used as an electronic device, and the electrical signal is transmitted in and out of the molecules via a couple of contact electrodes. This experiment’s architecture consists of a single-molecule junction based on a metal–molecule–metal structure (Aradhya and Venkataraman, 2013).

In light of this context, our research group proposed adjustment of the electron conductance in thiahelicenes by use of a customized synthetic approach. This modulation relies on the principles of quantum interference (QI) to achieve the desired effects (Lambert, 2015). Understanding QI is crucial for designing efficient molecular electronics components because the conductance of a tunneling electron through a π-conjugated molecule may be affected by the presence of different pathways in the orbital structure of the molecule. The concept of QI was originally adapted from the Aharonov–Bohm effect (Webb et al., 1985) to substituted benzenes (Sautet and Joachim, 1988; Hsu and Jin, 2009). Furthermore, Solomon et al*.* refined the concept in the context of molecular electronics, clarifying concepts such as constructive or destructive QI (Solomon et al., 2008a; Solomon et al., 2008b; Solomon et al., 2010). In the phase-coherent regime, the interference of the electron wave function flowing through the molecules’ constructive or destructive quantum interference (CQI and DQI, respectively) affects the molecular conductance (Zhang et al., 2013). It is now established that destructive QI leads to lower conductance in tunneling junctions. Single-molecule conductance measurements have been actively investigated using different methods such as the commonly used mechanically controlled break-junction (Reed et al., 1997) and scanning tunneling microscopy break-junction (STM-BJ) experiments (Xu and Tao, 2003). The conductance of the molecule is determined by analyzing the transmission function *T*(*E*) of a molecular junction, which represents the probability of electrons with energy *E* passing from one electrode to the other through the molecule. This characterizes the molecule’s ability to conduct electricity because mathematically, the conductance (*G*) is proportional to the transition function. Energy (*E*) is usually considered relative to the system Fermi energy, (*E*_F_). In addition, sharp resonances in the transmission curve correspond to the energy of molecular orbitals, such as the highest occupied molecular orbital (HOMO) and the lowest unoccupied molecular orbital (LUMO), so it talks about constructive QI when the transmission curve is smooth and featureless between HOMO and LUMO resonance. In contrast, destructive QI leads to sharp anti-resonances in the transmission curve where *T*(*E*) approaches 0 (Sadeghi, 2018; Zotti and Leary, 2020).

**FIGURE 1:**
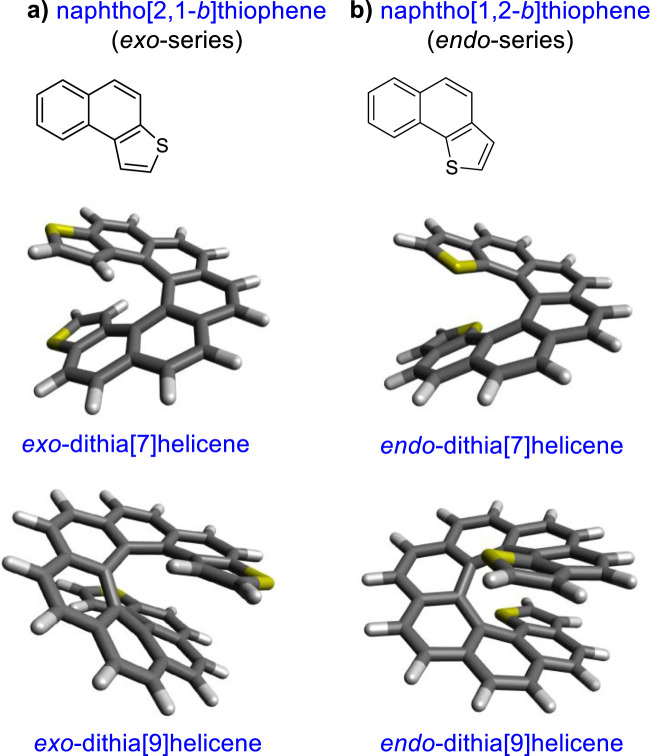
**(A)** *Exo-* and **(B)** *endo*-series, illustrated for dithia[7] and [9]helicenes, and details of the thiophene type of ring fusion endowing an *exo-* or an *endo-*arrangement of the sulfur atoms with respect to the helix (top left insets).

Recently, the conductance of molecules containing five-member heterocycles, another research interest, was also revealed to be related to QI. The effect of aromaticity and connectivity on the conductance of a five-membered ring was calculated by density functional theory (DFT), and in thiophene-like molecules, the conductance has been correlated with either aromaticity of the ring or electronegativity of the atom depending on the connection (Borges and Solomon, 2017; Chen et al., 2014). The concept that increasing aromaticity decreases molecular conductance was proposed by Breslow and Foss (2008). More recently, research studies revealed that the negative relationship between conductance and molecular aromaticity is not observed in all of the configurations of the molecular junction based on expanded porphyrins. In the case of the porphyrins, the results demonstrated that the phase and amplitude of the HOMO and LUMO play a major role in determining the fundamental aspects of the molecular conductance of π-conjugated molecules (Stuyver et al., 2018a; Stuyver et al., 2018b). Furthermore, analysis of different benzodithiophene (BDT) derivatives demonstrated that the conductance of a tunneling junction depends on the position and depth of a QI, both of which can be controlled synthetically (Zhang et al., 2013; Zhang et al., 2014). With this objective in mind, we set up a synthetic strategy to obtain a series of thiophene-fused helicenes with a well-defined positioning of the sulfur atom (9). Figure 1 depicts the structures of these molecules, dithia[n]helicenes (DTH), which display an *exo* or *endo* sulfur topology with respect to the helical structure. In more precise terms it denotes a naphtho[2,1-*b*]thiophene or naphtho[1,2-*b*]thiophene type of ring fusion of both terminal thiophenes, respectively, which we propose controls the emergence of conducting, linearly conjugated, as well as disrupted, cross-conjugated pathways.

In this work, we relate the electronic transport through our molecules to three key variables: a) the *exo-*/*endo-*topology of the S atom within the ring; b) the parity (*odd/even*) of the overall number of rings conforming to the helicene; and c) the size of the circuit. These series rank from [7] to [11] fused rings, having both *exo-*, *endo-*, or mixed topology. We explain next how these variables may exert a control on the electronic flow through the molecule.

### 1.1 Bond topology control: the role of topology, parity, and size in controlling the flow of electrons in dithia[n]helicenes

**FIGURE 2:**
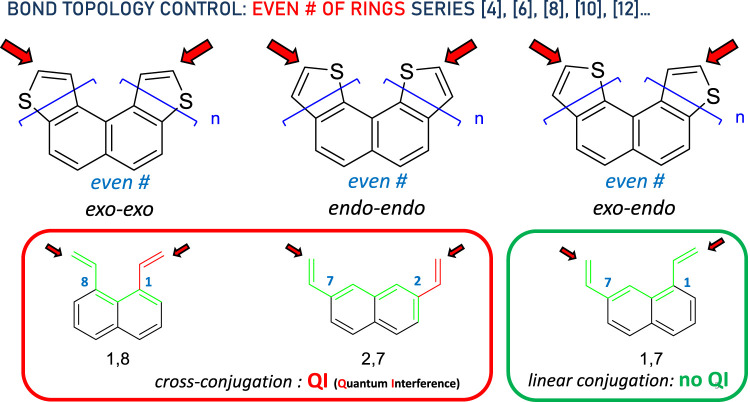
Bond topology control in the DTH series with an *even* # of rings. Red arrows indicate binding spots to the electrodes. In the bottom, the π system has been simplified, displaying either cross- (red circuit) or linear conjugation (green circuit), consistent with the presence or absence of quantum interference, respectively, between both ends.

For an *even* number of rings ([4], [6], [8], [10], [12]…), regardless of the size of the DTH, the bond topology is similar to that shown in Figure 2. The three types of thiophene ring fusions are shown at the top of the diagram and correspond to *exo*–*exo*, *endo*–*endo*, and *exo*–*endo* topologies. If we assume bonding through the a-carbon (represented by the red arrows), the three types of conjugated systems may be further simplified to the circled structures. For n *even*, *exo*–*exo*-[n]DTH reduces to a *cross-conjugated* π system exemplified as 1,8-divinylnaphthalene, and so does *endo*–*endo*-[n]DTH to 2,7-divinylnaphthalene, in which disrupted conjugation has been represented by a green circuit ending in a red one. Conversely and still for n *even*, *exo*–*endo*-[n]DTH reduces to a *linear conjugated* π system exemplified as 1,7-divinylnaphthalene, in which efficient conjugation has been represented by a green circuit connecting both ends.

**FIGURE 3:**
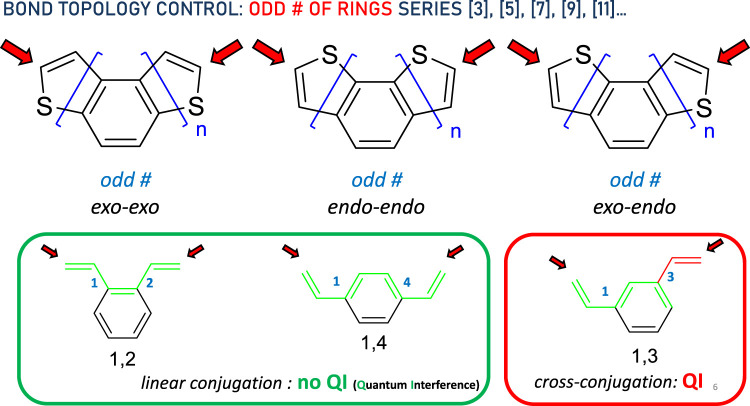
Bond topology control in the DTH series with an *odd* # of rings. Red arrows indicate binding spots to the electrodes. In the bottom, the π system has been simplified, displaying either linear (green circuit) or cross-conjugation (red circuit), consistent with the absence or presence of quantum interference, respectively, between both ends.

Next, we consider the *odd* number of rings. A complete symmetric pattern emerges here. In this case, again regardless of the DTH size ([3], [5], [7], [9], [11]…), the bond topology is represented in Figure 3. The three types of thiophene ring fusions displaying *exo*–*exo*, *endo*–*endo*, and *exo*–*endo* topologies are shown at the top of the diagram. These types of conjugated systems may be further simplified to the circled structures. For n *odd*, *exo*–*exo*-[n]DTH reduces to a *linear-conjugated* π system exemplified as 1,2-divinylbenzene, and so does *endo*–*endo*-[n]DTH to 1,4-divinylnaphthalene, in which efficient conjugation has been represented by a green circuit from end to end. Conversely, *exo*–*endo*-[n]DTH reduces to a *cross-conjugated* π system exemplified as 1,3-divinylbenzene, in which disrupted conjugation has been again represented by a green circuit ending in a red one.

In short, we can draw expected circuits of conductance originated by the bond topology. Linear conjugation facilitates efficient electron delocalization, enabling π-electrons to flow freely along the entire length of the π system, from end to end, as depicted by the green circuit between the red arrows. On the contrary, cross-conjugation disrupts this process, disabling π-electrons from flowing freely between both ends.

The synthetic strategies used for preparing these series of dithia[n]helicenes and a theoretical study of the binding properties of these molecules to a gold electrode are presented.

## 2 Materials and methods

### 2.1 Synthesis of *exo*- and *endo*-dithia[10] and [11]helicenes

**SCHEME 1:**
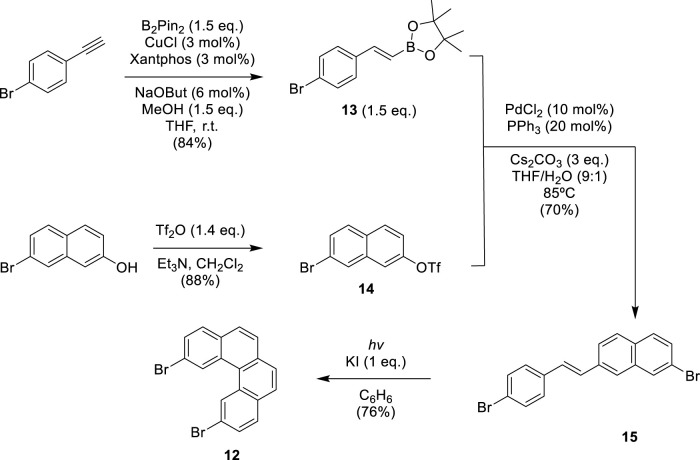
Synthesis of central fragment 2,11-dibromobenzo[*c*]phenanthrene **12.**

**SCHEME 2:**
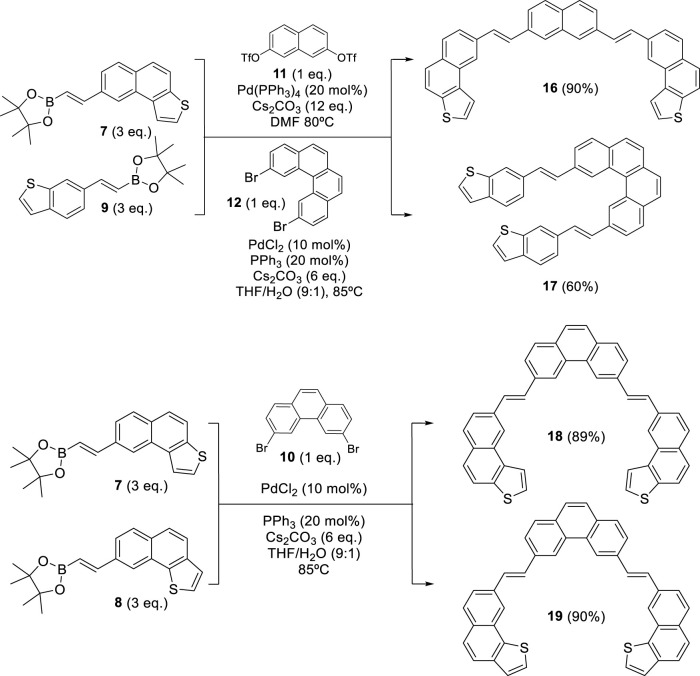
Synthesis of *bis*-(*E*)-stilbenic precursors **16, 17, 18**, and **19** through a double Suzuki coupling reaction.

In previous works, our group proposed a modular synthetic route using different fragments to synthesize *exo*- and *endo-*dithia[7] and [9]helicenes. (9) Furthermore, by STM, the self-assembly on the gold surface of these molecules was analyzed to observe one single molecule in the case of the *exo*-compound, exposing its electrical conductance and even allowing visual chiral assignment (Baciu et al., 2020). The *odd* number of ring series was later expanded using a similar modular assembly to dithia[9]helicenes, also with the *exo*- and *endo*-topology on both ends (Baciu et al., 2022). With the development of these new enlarged helicenes, interesting photophysical and chiroptical properties arise, such as significant mirror-image CPL activity, ranking among the largest reported for a helicene derivative. This established our modular synthetic protocols as reliable and robust, enough to face new and more demanding challenges. This modular synthetic pathway relies on a final step of photochemical reaction to create armchair-type ring fusions between specific variable-sized fragments. These fragments can occupy either a central or a terminal position. (9) The fragments used as central building blocks, 3,6-dibromophenanthrene **10**, naphthalene-2,7-diyl*bis*(trifluoromethanesulfonate) **11**, and 2,11-dibromobenzo[c]phenanthrene **12**, were prepared using different synthesis strategies (see Schemes 1, 2). The synthesis method of **10** was reported by our group in previous work (Baciu et al., 2021). Fragment **11** was easily obtained from 2,7-dihydroxynaphthalene by double trifluoromethanesulfonation using triflic anhydride (see SI). Central fragment **12** was obtained through oxidative photocyclization, as described in Scheme 1. Hydroboration of 4-bromoethynylbenzene provided the vinylboronic derivative **13** needed for the subsequent Suzuki coupling with **14**. The stilbenic precursor **15** obtained this way was irradiated with UV light using the LED technology to afford **12**.

For the synthesis of the terminal fragments, a route consisting of a Sonogashira reaction and a hydroboration was used to obtain fragments **7** and **8**. These contain the specific naphtho[2,1-*b*]thiophene and naphtho[1,2-*b*]thiophene moieties, respectively, ready to be assembled into central fragments by Suzuki coupling (Baciu et al., 2020). Furthermore, the smaller benzo[*b*]thiophene derivative **9** was obtained through analogous synthetic pathways (Baciu et al., 2022).

With these fragments in hand, our synthetic pathway adapted state-of-the-art methods for the palladium-catalyzed coupling reaction with the pioneering work on the photochemical synthesis of thiahelicenes, which was developed by Wynberg and Groen (1968), which inspired the key steps of this synthetic method. All (*E*)-stilbenic precursors were obtained by using the Suzuki coupling reaction using vinylboronic esters **7–9** and the different central fragments described above (Scheme 2).

**SCHEME 3:**
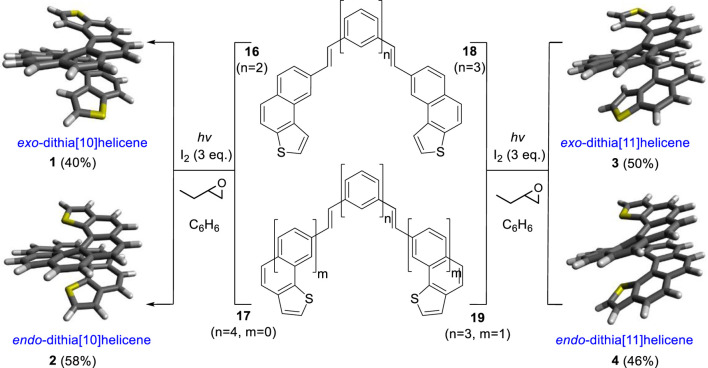
Synthesis of dithiahelicenes **1**, **2**, **3**, and **4** *via* double-oxidative photocyclization of *bis*-(*E*)-stilbenic precursors **16**, **17**, **18**, and **19**. n and m represent the overall number of fused armchair rings.

Then, oxidative photocyclization (Jørgensen, 2010) of the *bis*-stilbenic precursors **16**, **17**, **18,** and **19** under Mallory–Katz conditions was the last synthetic step required. It was done by use of 365 nm LED irradiation in benzene to improve the solubility of the substrate, under an Ar atmosphere, with iodine as the final oxidizer and 1,2-butylene oxide as a HI scavenger. This afforded *exo*-dithia[10]helicene **1**, *endo*-dithia[10]helicene **2**, *exo*-dithia[11]helicene **3**, and *endo*-dithia[11]helicene **4** in fair yields (Scheme 3).

### 2.2 Synthesis of dithiahelicenes of mixed topology: *exo*-*endo*-dithia[7] and [10]helicenes

**SCHEME 4:**
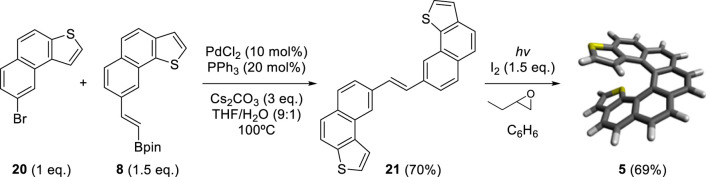
Synthesis of *exo–endo*-dithia[7]helicene **5**.

**SCHEME 5:**
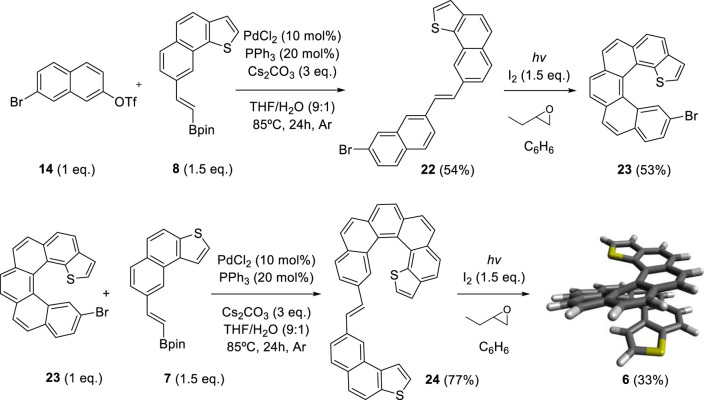
Synthesis of *exo*–*endo*-dithia[10]helicene **6**.

By adapting the synthetic strategy explained before, our group decided to synthesize dithiahelicenes with both an *exo* and an *endo* sulfur atom in the same molecule or mixed topology. *Exo*-*endo*-dithia[7]helicene **5** and *exo*-*endo*-dithia[10]helicene **6** were the selected candidates. In both syntheses, fragment **8** was used to introduce the *endo* thiophene terminal moiety. In the first case, an (*E*)-stilbenic precursor was obtained by coupling **8** and **20** (synthesized in previous work) (9) under typical Suzuki conditions (Scheme 4). In the second case, using the same principles, our group prepared fragment **23**. Subsequent coupling of **23** with **7** under Suzuki reaction conditions provided the (*E*)-stilbenic precursor ready for the final photocyclization. In both cases, the final helicenes were obtained by UV irradiation under Mallory–Katz conditions by using an LED source (Scheme 5).

### 2.3 Theoretical methods

Calculations were performed with Kohn–Sham density functional theory (DFT) and time-dependent DFT (TDDFT) linear response methods. Ground-state equilibrium structures were optimized with spin-restricted DFT. All structures were characterized as minima via harmonic vibrational frequency calculations. For the binding energies (Δ*E*_*f*_ (kcal/mol), DFT calculations were done at the B3LYP/6-311G(d,p) level for the organic moiety (C, H, and S) and LANL2DZ for heavy atoms (Au_10_ cluster). For accuracy, the basis set superposition error (BSSE) was taken into account. Two interacting fragments, dithiahelicene (fragment 1) and Au_10_ (fragment 2), were defined. First, both fragments were allowed to interact until minimum energy was located by optimization of the complex and corroborated by vibrational analysis. Second, we optimized the isolated fragments, placed them in the complex orientation, and separated them along the direction of the bond from the equilibrium binding distance until a negligibly small interaction was found, and further away (typically up to 20 Å of separation), evaluating at this point the energy by a single-point calculation. A counterpoise calculation of these unbound complexes was carried out to corroborate the accuracy of the calculation (confirming a BSSE = 0). The binding energy was then obtained by subtracting both calculated electronic energies. For the dithiahelicene fragment, full optimization afforded *C*_2_ symmetries for the *exo* and *endo* cases and *C*_1_ for the mixed *exo*–*endo* configuration. The gold tip (Au_10_) was taken from the bulk fcc gold crystal (*F*m−3m) and relaxed at the theory level used for gold (B3LYP/LANL2DZ) while imposing *T*_d_ symmetry as a constraint. Then, it was used as a rigid fragment. For the optical gaps (TDDFT) and HOMO-LUMO (DFT) calculations, geometry optimizations and vibrational analysis were performed with the CAM-B3LYP functional and the def2SVPP basis set. This work was done using Gaussian 16 version C.01 (G16) (Frisch et al., 2022).

## 3 Results and discussion

### 3.1 DFT calculation of the binding properties to gold

**FIGURE 4:**
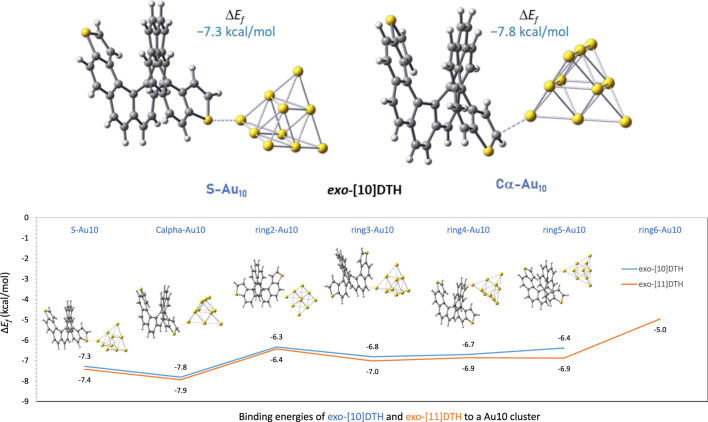
On the top, details of the two preferred binding configurations of an Au_10_ gold tip interacting at the sulfur and at the α-carbon position using *exo*-dithia[10]helicene as a representative model. On the bottom: binding energies, Δ*E*_*f*_ (kcal/mol), of an Au_10_ gold tip interacting with the different rings of *exo*-dithia[10] and [11]helicenes. Given as energies of formation of the complex by DFT calculations, at the B3LYP/6-311G(d,p) on C, H, S, and LANL2DZ on Au after BSSE correction.

Our group analyzed the binding energy of two of the largest dithiahelicenes available both in *exo-* and *endo-*configurations, namely, dithia[10]helicenes and dithia[11]helicenes ([10] or [11]DTH) to a gold tip, by DFT calculation. The tip consisted of 10 gold atoms forming a tetrahedron (*T*_d_ symmetry), with its vertex pointing in the (1, 1, 1) direction of a standard *fcc* gold crystal. Figure 4 shows the most significant results obtained for these molecules. Focusing on the *exo*-isomer, several general features are revealed. First, as anticipated, there is a preference for the terminal thiophene ring when binding to the Au_10_ cluster. Binding at the inner carbo-rings (rings 2–5 or 6) is less favorable for approximately 1–1.5 kcal/mol, with these having binding energies comparable to those of homologous carbo[n]helicenes. The favored position within the terminal ring is the α-carbon position, slightly favored over the sulfur atom by approximately 0.5 kcal/mol. A significant degree of pyramidalization is evident at the α-carbon during binding at this position. These binding energy patterns are reproducible and comparable regardless of the parity (*odd* or *even* number of rings) of the helicene. There is little difference in the binding of the tip at the inner rigs (rings 2–5), which always occurs in an *η* ^2^ manner, i.e*.*, involving a pair of CH carbons of the outer helix, while for α-C or S binding in the thiophene, the first ring is always *η* ^1^. For the *exo*-[11]DTH, it is difficult for the tip to fit snugly into the innermost ring (ring 6) because of crowding, resulting in reduced binding energy.

For the *endo-*isomers, the binding scenario is less meaningful and its analysis more intricate, resulting in a less straightforward picture compared to that of the *exo-*isomer. Distortion of the initial thiophene ring, caused by a bigger *endo*-sulfur atom, leads to a variety of configurations where a portion of the gold cluster’s edge rests on the ring’s surface. This results in unusually large binding energies as more than one gold atom of the cluster cooperatively binds to the helicene surface. However, in instances where the tip is attached via a single gold atom (*η* ^1^), the binding energies are similar to those of the *exo-*isomer, which is a good model of the interaction. The complete array of configurations and their respective binding energies are detailed in SI.

These data suggest several things. First, thiahelicenes are preferably grafted to the gold electrode through the α-carbon, rather than to the sulfur atoms as initially anticipated. This is in line with our study findings, shown in Figures 2, 3 with a red arrow. Still, the small difference in binding energies (0.5–1.5 kcal/mol) restrains the possibility of displaying selective binding (through the α-carbon) only to experiments conducted at very low temperatures. Second, in absolute terms, binding energies are relatively small, and it is likely the possibility of a complex scenario of multiple binding sites acting cooperatively on a gold surface, so dissecting a single-atom contact in a break-junction experiment may be extremely difficult. That makes the actual experiment quite intricate and challenging to perform. Attempts to measure conductance in such molecules at room temperature have afforded unexpectedly poor results (de Ara et al., 2024), presumably in our opinion due to, in addition to all of the above, thermal molecular jiggling between the electrodes. We hope that future, more accurate low-temperature experiments may allow us to differentiate between the different binding topologies reported in this article.

### 3.2 Calculated molecular HOMO-LUMO energies and optical gaps of the dithia[n]helicene series

**FIGURE 5:**
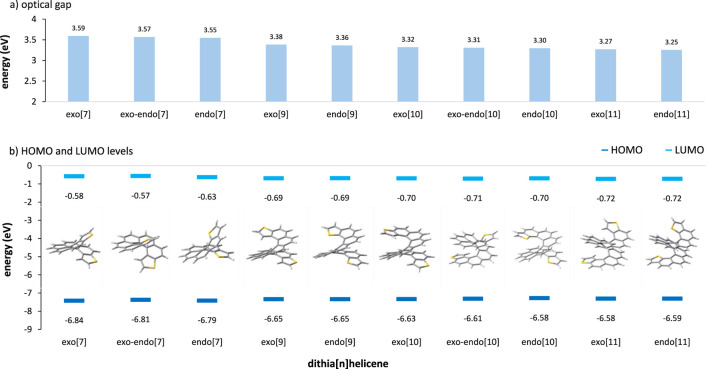
**(A)** TDDFT-calculated optical gap and **(B)** DFT-calculated HOMO and LUMO energies of the DTH series covered in this work. From CAM-B3LYP/def2SVPP at the ground-state optimized geometry, *C*_2_ for *exo-* and *endo*-DTH and *C*_1_ for *exo–endo*-DTH.

Now, a final note must be made to assess the effect of DTH size within the same parity series. The interface coupling between the molecule and the electrode greatly affects the conducting behavior in conjugated molecular systems. This interaction is governed by many factors, such as the HOMO-LUMO gap and the alignment of this gap to the Fermi energy level of the electrode (Liu et al., 2009; Wang et al., 2009). For maximizing the electron transmission, a certain degree of alignment between the molecular orbital levels and the Fermi level is required (Xin et al., 2019). For completeness, we report here a DFT calculation of the molecular orbitals usually dominating the conductance, which are the HOMO and the LUMO, as well as the optical gap of all the DTHs covered in this work (Figure 5).

The optical gap of a molecule corresponds to the energy of the lowest electronic transition accessible via absorption of a single photon and accounts for the stabilization energy of the electron–hole pair created during the transition (Bredas, 2014). This leads to smaller and usually more reliable gaps compared to the gross estimate provided by DFT HOMO-LUMO gaps. In our systems, similar to other conjugated molecules, the optical gap decreases with increasing molecular size. However, this effect is not very pronounced here, i.e., less than a tenth of an eV (*ca*. 0.08 eV) per added ring, displaying a similar value for all the series (*exo*, *exo–endo*, or *endo*). For a given size, differences in the optical gap are equally very small, following the order *exo* > *exo–endo* > *endo*. This gap is a crucial parameter in determining the conductance of molecular wires since it is directly correlated to the barrier that must be crossed by the electrons in the tunneling process (Liu et al., 2008).

## 4 Conclusion

In this article, we report a modular synthetic route suitable to prepare DTH of sizes ranging from [7] to [11] using our cutting-edge methods of Pd-catalyzed coupling reactions, followed by an LED-driven final step of photocyclization. These molecules exhibit either *exo*, *endo*, or a yet unreported mixed *exo–endo* configuration, indicating the S atom arrangement of the terminal thiophene fused rings at both ends. By examining the bond topology of these compounds more closely, we uncovered interesting properties. The expected electronic transport through them depends on three key variables: a) the *exo-*/*endo-*topology of the S atom within the ring; b) the parity (*odd*/*even*) of the total number of rings that form the helicene; and, to a less extent, c) the size of the circuit. Expressed in chemical terms, this is the result of the occurrence of either 1) cross-conjugation between the α-carbons grafted to the gold electrodes, which is associated with the occurrence of QI phenomena, or 2) linear conjugation between the same anchoring positions, corresponding to the absence of QI phenomena, expressed in physical terminology. Next, the binding properties to an Au_10_ cluster of four representative DTH featuring *exo-* and *endo-*topologies, as well as *even* [10] and *odd* [11] number of rings, were calculated by DFT. This study revealed that the terminal thiophene was the most favored ring for binding, and within this ring, the α-carbon was identified as the preferred binding site, exhibiting a stability of approximately 0.5 kcal/mol, greater than that of sulfur atom binding. By comparing similar DTH with adjustable HOMO/LUMO energies, we aim to distinguish the crucial factor of bond topology from other electronic factors, thereby paving the way for the exploration of QI in helically conjugated molecules. The next stage of this work will convey the experimental measure of actual conductance using gold electrodes, taking into account that only very low-temperature, meticulous experiments might unveil the quantum interference phenomena described in this study.

## Data Availability

The original contributions presented in the study are included in the article/Supplementary Material; further inquiries can be directed to the corresponding author.
