# Supplementary material for: Design and synthesis of thiahelicenes for molecular electronics
Source: Front Chem. 2024 Oct 14;12:1471413. doi: 10.3389/fchem.2024.1471413 (PMC11513318; doi:10.3389/fchem.2024.1471413)
Supplement: Supplementary file 1 [file DataSheet1.pdf]

Supplementary Material

Design and Synthesis of Thiahelicenes for Molecular Electronics

Bianca C. Baciu[1†], Pawel J. Bronk[1†], Albert Guijarro[1*]

[1]Instituto Universitario de Síntesis Orgánica and Departamento de Química Orgánica, Campus de San Vicente del Raspeig, Universidad de Alicante, Alicante, Spain

*** Correspondence:**
Albert Guijarro: aguijarro@ua.es

Keywords: organic synthesis, dithiahelicene, photocyclization, bond topology, molecular solenoid, DFT binding energy, gold electrode

Contents

1. Supplementary Data 4

1.1. General Methods *4*

1.2. Photochemistry *4*

1.3. Synthesis and characterization of compounds *4*

1.3.1. Naphthalene-2,7-diyl bis(trifluoromethanesulfonate) 4

1.3.2. (E)-2-(4-Bromostyryl)-4,4,5,5-tetramethyl-1,3,2-dioxaborolane 5

1.3.3. 1.4-bromonaphthalen-2-yl-trifluoromethanesulfonate 5

1.3.4. *(E)*-2-bromo-7-(4-bromostyryl)naphthalene 6

1.3.5. 2,11-dibromobenzo[c]phenanthrene. 6

1.3.6. (*E*)-8-(2-(7-bromonaphthalen-2-yl)vinyl)naphtho[1,2-*b*]thiophene 7

1.3.7. 14-bromonaphtho[1',2':5,6]phenanthro[4,3-*b*]thiophene 7

1.3.8. (*E*)-8-(2-(naphto[2,1-*b*]thiophen-8-yl)vinyl)naphto[1,2-*b*]thiophene 8

1.3.9. (*E*)-14-(2-(naphtho[2,1-*b*]thiophen-8-yl)vinyl)naphtho[1',2':5,6]phenanthro[4,3-*b*]thiophene9

1.3.10. 2,7-bis((E)-2-(naphtho[2,1-b]tiophen-8-yl)vinyl)naphthalene 9

1.3.11. 2,11-bis((E)-2-(benzo[b]tiophen-6-yl)vinyl)benzo[c]phenanthrene 10

1.3.12. 3,6-bis((E)-2-(naphtho[2,1-b]tiophen-8-yl)vinyl)phenanthrene 10

1.3.13. 3,6-bis((E)-2-(naphtho[1,2-b]tiophen-8-yl)vinyl)phenanthrene 11

1.3.14. 1,14-dithia[7]helicene or *exo-endo*-dithia[7]helicene 11

1.3.15. 1,20-dithia[10]helicene or *exo-endo*-dithia[10]helicene 12

1.3.16. 3,20-Dithia[10]helicene or *exo*-dithia[10]helicene 12

1.3.17. 1,22-Dithia[10]helicene or *endo*-dithia[10]helicene 12

1.3.18. 3,22-Dithia[11]helicene or *exo*-dithia[11]helicene 13

1.3.19. 1,24-Dithia[11]helicene or *endo*-dithia[11]helicene 13

1.4. ^{1}H-NMR and ^{13}C-NMR spectra of compounds *14*

1.4.1. Naphthalene-2,7-diyl bis(trifluoromethanesulfonate) (300 Mhz, $CDCl_3$) 14

1.4.2. (*E*)-2-(4-Bromostyryl)-4,4,5,5-tetramethyl-1,3,2-dioxaborolane (300 MHz, $CDCl_3$) 15

1.4.3. 4-bromonaphthalen-2-yl trifluoromethanesulfonate (300 MHz, $CDCl_3$) 16

1.4.4. *(E)*-2-bromo-7-(4-bromostyryl)naphthalene (400 MHz, $CDCl_3$) 17

1.4.5. (*E*)-8-(2-(7-bromonaphthalen-2-yl)vinyl)naphtho[1,2-*b*]thiophene (400 MHz, $CDCl_3$) 17

1.4.6. 2,11-dibromobenzo[c]phenanthrene (300 MHz, $CDCl_3$) 18

1.4.7. (*E*)-14-(2-(naphtho[2,1-*b*]thiophen-8-yl)vinyl)naphtho[1',2':5,6]phenanthro[4,3-*b*]thiophene (400 MHz, $CDCl_3$) 19

1.4.8. 14-bromonaphtho[1’,2’:5,6]phenanthro[4,3-*b*]thiophene (400 MHz, $CDCl_3$) 20
1.4.9. 1,14-dithia[7]helicene or *endo*-*exo*-dithia[7]helicene (400 MHz, $CDCl_3$) 21
1.4.10. 1,20-dithia[10]helicene or *endo*-*exo*-dithia[10]helicene (400 MHz, $CDCl_3$) 22
1.4.11. 3,20-Dithia[10]helicene or *exo*-dithia[10]helicene (300 MHz, $CDCl_3$) 23
1.4.12. 1,22-Dithia[10]helicene or *endo*-dithia[10]helicene (300 MHz, $CDCl_3$) 24
1.4.13. 3,22-Dithia[11]helicene or *exo*-dithia[11]helicene (300 MHz, $CDCl_3$) 25
1.4.14. 1,24-Dithia[11]helicene or *endo*-dithia[11]helicene (300 MHz, $CDCl_3$) 26
2. Supplementary Figures and Tables 27
Supplementary Figures 1. 27
Supplementary Figures 2. 27
Supplementary Figures 3. 28
Supporting Figure 4. 29
3. Supporting Table 1. DFT CALCULATIONS OF THE BINDING PROPERTIES TO GOLD 31
Exo-dithia[10]helicene-Au_{10} 31
Endo-dithia[10]helicene-Au_{10} 33
Exo-dithia[11]helicene-Au_{10} 35
Endo-dithia[11]helicene-Au_{10} 37
Overall view of ΔE_f *(kcal/mol) of exo- and endo-dithia[10] and [11]helicene-*Au_{10} *complexes* 39

1. Supplementary Data

1.1. General Methods

Commercially starting materials and solvents for photochemistry, chromatography and recrystallization were used without further purification, unless otherwise stated. Commercially unavailable reagents were synthesized via different methods that will be explained separately.

Gas chromatography analyses (GLC) were carried out with a Hewlett Packard HP-5890 instrument equipped with a flame ionization detector and a 30 m HP-5 capillary column (0.32 mm diam, 0.25 µm film thickness), using nitrogen as carrier gas (12 psi). Column chromatography was performed with Merck silica gel 60 (0.040-0.063 µm, 240-400 mesh). Thin-layer chromatography (TLC) was performed on precoated silica gel plates (Merck 60, F254, 0.25 mm). TLC detection was done by UV_{254} light, R_f values are given under these conditions. NMR spectra were recorded on a Bruker Avance 300 and Bruker Avance 400 (300 and 400 MHz for ^{1}H-NMR, and 75 and 100 MHz for ^{13}C-NMR respectively) using $CDCl_3$ as a solvent and TMS as internal standard. Chemical shifts (δ) are given in ppm vs. TMS. Infrared (IR) analysis was performed with a JASCO FT/IR 4100 spectrophotometer equipped with an ATR component. LRMS were performed using the electron impact (EI) mode at 70 eV in an AGILENT 5973N mass spectrometer coupled with an AGILENT 6890N gas chromatographer. Melting points were performed with a Reichert Thermovar polarizing light microscope and melting points apparatus and have been corrected.

1.2. Photochemistry

Two 50 W LED boards were fixed to the walls of a vertical aluminum cylinder (15 cm diameter × 25 cm height) facing each other on opposite sides and connected to the corresponding power units. Small heat dissipaters provided with fans were attached to the LED boards to prevent overheating. This constituted the irradiation chamber. A 250 mL Schlenk single-walled borosilicate tube was placed in the middle of this chamber containing the reaction mixture and was irradiated from opposite sides, at ca. 4 cm distance from each LED plate, while being magnetically stirred from the bottom. With this setup, we worked without reflux and no additional refrigeration of the central reaction tube was needed. We use cyclohexane or benzene as solvents. For the degassed reaction, Ar was mildly bubbled through the reaction as explained above.

1.3. Synthesis and characterization of compounds

1.3.1. Naphthalene-2,7-diyl bis(trifluoromethanesulfonate)[1]

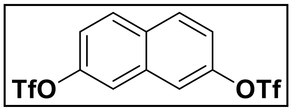

This compound was prepared by the procedure described in the literature.[2] In a 100mL round flask, 2,7-dihydroxinaphtalene (1.63 g, 10 mmol, 1eq.) was added and then the flask was sealed with septum and three cycles vacuum/argon were then performed. Then 32 mL of dry CH_2Cl_2 and 6.4 mL of dry Et_3N were added.

---

[1] Katz, T. J.; Liu, L. B.; Willmore, N. D.; Fox, J. M.; Rheingold, A. L.; Shi, S.; Nuckolls, C.; Rickman, B. H. (1997) An Efficient Synthesis of Functionalized Helicenes J. Am. Chem. Soc. 119, 10054-10063. DOI: 10.1021/ja9721327

[2] Hacker, A. S.; Pavano, M.; Wood, J. E.; Immoos, C. E.; Hashimoto, H.; Genis, S. P.; Frantz, D. K. (2018) Synthesis and Electronic Properties of Fluoreno[2,1-*a*]fluorenedione and Fluoreno[1,2-*a*]fluorenedione J. Org. Chem. 83, 510-515. DOI: 10.1021/acs.joc.7b02699

The solution was cooled to 0 ℃ over an ice bath. Triflic anhydride (4 mL, 24 mmol, 2.4 eq.) was added drop by drop over 5 minutes while stirring. The dark reaction mixture was allowed to warm to room temperature and stirred for 20 h. After checking that there was not reagent, 10 mL of H_2O and 10 mL of CH_2Cl_2 were added to the mixture. The aqueous phase was extracted with CH_2Cl_2 (3 x 15 mL) and the combined organic phase was dried over Mg_2SO_4, it was filtered and concentrated in vacuo. The dark brown oil was then purified by column chromatography on silica gel (hexane-EtOAc 95:5) to afford a white solid in 83% of yield.

White solid; melting point: 51.9-54.0 ℃; R_f: 0.37 (hexano:EtOAc 9:1). ^{1}H-RMN (300 MHz, $CDCl_3$) δ= 8.00 (d, J = 9.2 Hz, 2H), 7.81 (d, J = 2.4 Hz, 2H), 7.48 (dd, J = 9.1, 2.4 Hz, 2H) ppm; ^{13}C-RMN (101 MHz, $CDCl_3$) δ= 148.39 (-CO-, 2C), 133.74 (-C-, 1C), 131.41 (-C-, 1C), 130.96 (-CH- 2C), 121.28 (-CH-, 2C), 119.64 (-CH-, 2C), 118.91 (q, $^1J_{C-F}$ = 320.8 Hz, -CF-, 2C); MS (EI, 70 eV): m/z 424.1 (M^+92), 291.1 (100), 263.1 (47), 199.1 (49) 130.1 (86), 102.1 (53), 69.1 (23); IR (neat) ν_{max}: 1413, 1210, 1134, 1111, 957, 883, 849, 728 cm^{-1}.

1.3.2. **(E)-2-(4-Bromostyryl)-4,4,5,5-tetramethyl-1,3,2-dioxaborolane**[3]

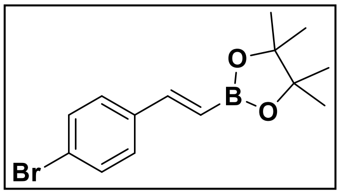

The compound was prepared by adapting a procedure from the literature.[4] In an oven-dried 100 mL Schlenk tube, CuCl (5.93 mg, 0.06 mmol, 0.03 eq.), NaO*t*-Bu (11.53 mg, 0.12 mmol, 0.06 eq.) and Xantphos ligand (34.71 mg, 0.06 mmol, 0.03 eq.) were added. Then, after 3 cycles of vacuum/argon, 1 mL of dry THF was injected and the solution was stirred for 30 minutes at room temperature. Then bis(pinacolato)diboron (761.82 mg, 3 mmol, 1.5 eq.) dissolved in 1.5 mL of dry THF, were added and the reaction mixture was stirred for 10 minutes at room temperature. Finally, 1-bromo-4-ethynylbenzene (362.06 mg, 2 mmol, 1 eq.) dissolved in 1 mL of dry THF was added, followed by dry MeOH (0.121 mL, 3 mmol, 1.5 eq.). The reaction mixture was stirred at room temperature until no starting material was detected by TLC (3 hours). After 3 hours the reaction mixture was filtered through a pad of celite, and the residue was purified by column chromatography on silica gel (hexane-EtOAc 98:2) obtaining a yellow wax in 84% yield.

Yellow wax; R_f= 0.33 (hexane-EtOAc 98:2); ^{1}H-NMR ($CDCl_3$, 300 MHz): δ= 7.49- 7.42 (m, 2H), 7.37-7.27 (m, 3H), 6.14 (d, J = 18.4 Hz, 1H), 1.31 (s, 12H) ppm. ^{13}C-NMR ($CDCl_3$, 75 MHz): δ= 148.20 (-CH-, 1C), 136.52 (-C-, 1C), 131.89 (-CH-, 2C), 128.64 (-CH-, 2C), 123.04 (-C-, 1C), 83.61 (Pin-C-, 2C), 24.94 (Pin-CH3, 4C) ppm. MS (EI) *m/z*: 310.20 (M^++2, 71.7), 308.20 (M^+, 72.5), 295.15 (30.1), 293.10 (30.4), 224.10 (52.3), 222.10 (50.9), 210.10 (99.7), 208.10 (100), 194.10 (24.1), 192.10 (24.6), 143.20 (97.1), 129.20 (78.9), 102.20 (16.9), 77.20 (32.9). IR (neat) ν_{max}: 2977, 2931, 1628, 1485, 1346, 1323, 1269, 1211, 1142, 995, 972, 852, 802 cm^{-1}.

1.3.3. **1.4-bromonaphthalen-2-yl-trifluoromethanesulfonate**

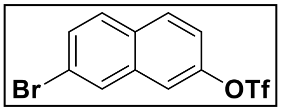

This compound was prepared by adapting a procedure from the literature.[4] In a 250 mL round flask, 7-bromonaphthalen-2-ol (2.23 g, 10 mmol, 1 eq.) was added and then the flask was sealed with septum and three cycles vacuum/argon were then performed. Then 64 mL of dry CH_2Cl_2 and 12.8 mL of dry Et_3N were added. The solution

---

[3] Wu, Z.-L.; Lan, X.; Gao, N.; Kang, X.; Wang, Z.; Hu, T.; Zhao, B. (2021) Highly efficient hydroboration of alkynes catalyzed by porous copper-organic framework under mild conditions J. of Catalysis. 404, 250-257. DOI: 10.1016/j.jcat.2021.09.033

[4] Lee, J.-E.; Kwon, J.; Yun, J. (2008) Copper-catalyzed addition of diboron reagents to α,β-acetylenic esters: efficient synthesis of β-boryl- α,β-ethylenic esters Chem. Commun. 6, 733-734. DOI: 10.1039/B716697D

was cooled to 0 ℃ over an ice bath. Triflic anhydride (2.31 mL, 14 mmol, 1.4 eq.) was added drop by drop over 5 minutes while stirring. The dark reaction mixture was allowed to warm to room temperature and stirred for 20 h. After checking that there was not reagent, 10 mL of H_2O and 10 mL of CH_2Cl_2 were added to the mixture. The aqueous phase was extracted with CH_2Cl_2 (3 x 15 mL) and the combined organic phase was dried over Mg_2SO_4, it was filtered and concentrated in vacuo. The dark brown oil was then purified by column chromatography on silica gel (hexane-CH_2Cl_2 80:20) to afford a yellow oil in 88% yield.

Yellow oil; R_f= 0.52 (hexane-CH_2Cl_2 80:20); ^{1}H-NMR ($CDCl_3$, 300 MHz): δ= 8.03 (d, J = 1.5 Hz, 1H), 7.88 (d, J = 9.0 Hz, 1H), 7.74 (d, J = 8.8 Hz, 1H), 7.67-7.58 (m, 2H), 7.38 (dd, J = 9.0, 2.5 Hz, 1H). ^{13}C-NMR ($CDCl_3$, 101 MHz) 147.86 (-C-, 1C), 134.54 (-C-, 1C), 130.87 (-CH-, 1C), 130.82 (-CH-, 1C), 130.15 (-CH-, 1C), 129.60 (-CH-, 1C), 122.11 (-C-, 1C), 120.18 (-CH-, 1C), 118.52 (-CH-, 1C), 118.91 (q, $^1J_{C-F}$ = 320.8 Hz, -CF-, 1C). MS (EI) m/z 355.90 (M^++2, 52.1), 353.90 (M^+, 50.8), 223.00 (41.4), 221.00 (42.6), 195.00 (98.0), 193.00 (100), 114.10 (56.7), 88.10 (10.2), 69.10 (11.5). IR (neat) ν_{max} 1628, 1504, 1423, 1215, 1142, 957, 929, 895, 844, 760 cm^{-1}.

1.3.4. ***(E)*-2-bromo-7-(4-bromostyryl)naphthalene**

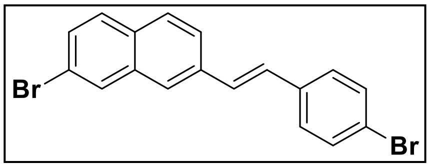

The compound was prepared by adapting to our substrate a Suzuki coupling described in the literature.[5] In an over-dried pressure tube were added $PdCl_2$ (17.33 mg; 0.1 mmol; 0.10 eq.), PPh_3 (52.46 mg; 0.2 mmol; 0.20 eq.), Cs_2CO_3 (977.46 mg; 3 mmol; 3 eq.), (E)-2-(4-bromostyryl)-4,4,5,5-tetramethyl-1,3,2-dioxaborolane (462.10 mg; 1 mmol; 1.5 eq.). The tube was sealed with a septum and after three cycles of vacuum/argon, 4-bromonaphthalen-2-yl trifluoromethanesulfonate (355.13 mg, 1 mmol, 1 eq.) was added dissolved in 3.6 mL of THF and 0.4 mL of H_2O were added with a syringe. After that the tube was closed and heated in an oil bath at 85℃ for 20 hours. An insoluble solid in suspension was observed in the tube. The insoluble solid, a green solid in 90% yield, was filtered and cleaned with H_2O and CH_2Cl_2.

Green solid; R_f= 0.42 (hexane-EtOAc 95:5); ^{1}H-NMR ($CDCl_3$, 400 MHz): δ= 7.99-7.97 (m, 1H), 7.79 (d, J = 8.5 Hz, 1H), 7.76-7.71 (m, 2H), 7.68 (d, J = 8.6 Hz, 1H), 7.55-7.48 (m, 3H), 7.42 (d, J = 8.5 Hz, 2H), 7.24 (d, J = 15.2 Hz, 1H), 7.16 (d, J = 15.2 Hz, 1H). MS (EI) m/z 390.05 (M^++4, 25.9), 388.00 (M^++2, 52.2), 386.05 (M^+, 26.9), 228.15 (100), 226.15 (55.1), 207.10 (25.1), 113.15 (36.6), 73.10 (10.3). IR (neat) ν_{max} 1616, 1577, 1485, 1396, 1068, 1003, 968, 910, 837, 706 cm^{-1}.

1.3.5. **2,11-dibromobenzo[c]phenanthrene.**[6]

---

[5] Molander, G. A.; Brown, A. R. (2006) Suzuki-Miyaura Cross-Coupling Reactions of Potassium Vinyltrifluoroborate with Aryl and Heteroaryl Electrophiles J. Org. Chem. 71, 9681-9686. DOI: 10.1021/jo0617013

[6] a) Parham, A. H.; Buesing, A.; Heil, H.; Stoessel, P. WO2010083869 A2 2010-07-29, German, CAplus. b) Sturm, L.; Artigas, A.; Coquerel, Y.; Bechtold, I. H.; Durola, F.; Bock, H. (2024) Helicene Aromaticity Deviates from the Clar Rule—On the Electronic Dissimilarity of Large Isomeric Fibonacenes Angew. Chem. Int. Ed. 63, e202403170. DOI: 10.1002/anie.202403170

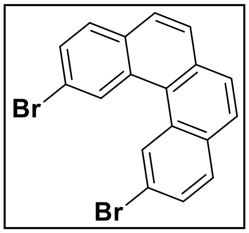

In an oven-dried 250 mL Schlenk tube were added (E)-2-bromo-7-(4-bromostyryl)naphthalene (38.81 mg, 0.1 mmol, 1 eq.) followed by 200 mL of benzene wee added. The mixture was stirred and heated a little bit to dissolve the reagent. After that potassium iodide (16.6 mg 0.1 mmol, 1 eq.) were added. The mixture was irradiated with LED's for 4 hours. After the reaction was completed, it was washed with aqueous $NaHSO_3$, dried under magnesium sulphate, filtered and the solvent evaporated under reduced pressure (15 Torr). The residue was purified by column chromatography on silica gel (hexane) to obtain a white solid in 76% yield.

White solid; R_f= 0.39 (hexane); ^{1}H-NMR ($CDCl_3$, 300 MHz): δ= 9.16 (d, *J* = 1.5 Hz, 2H), 7.86 (d, *J* = 8.6 Hz, 2H) 7.85 (d, *J* = 8.6 Hz, 2H), 7.81 (d, *J* = 8.6 Hz, 2H), 7.72 (dd, *J* = 8.6, 1.7 Hz, 2H). ^{13}C-NMR ($CDCl_3$, 101 MHz) 132.04 (-C-, 2C), 131.93 (-C-, 2C), 131.36 (-C-, 2C), 130.26 (-CH-, 2C), 129.85 (-CH-, 2C), 129.48 (-CH-, 2C), 127.80 (-CH-, 2C), 127.26 (-CH-, 2C), 125.35 (-C-, 2C), 121.81 (-C-2C). MS (EI) *m/z* 388.00 (M^++4, 48.2), 386.00 (M^++2, 95.2), 384.00 (M^+, 46.1), 226.20 (100), 224.20 (44.2), 113.10 (42.3), 112.20 (33.9). IR (neat) ν_{max} 2924, 2854, 1593, 1485, 1334, 1099, 1007, 957, 837, 702, 663 cm^{-1}.

1.3.6. (*E*)-8-(2-(7-bromonaphthalen-2-yl)vinyl)naphtho[1,2-*b*]thiophene

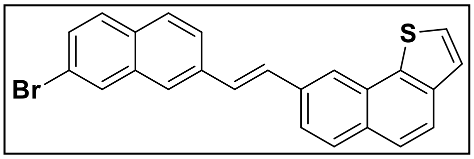

In a pressure tube, $PdCl_2$ (7.09 mg, 0.04 mmol, 0.1 eq.), PPh_3 (20.98 mg, 0.08 mmol, 0.2 eq.), Cs_2CO_3 (390.98 mg, 1.2 mmol, 3 eq.), 7-bromonaphthalen-2-yl trifluoromethanesulfonate (142.05 mg, 0.4 mmol, 1 eq.) and (*E*)-4,4,5,5-tetramethyl-2-(2-(naphtho[1,2-*b*]thiophen-8-yl)vinyl)-1,3,2-dioxaborolane (201.76 mg, 0.6 mmol, 1.5 eq.) were added. Then, the tube was sealed with a septum and three cycles of vacuum/argon were performed. THF (2.4 mL) and H_2O (0.27 mL) were added with purged syringed. The pressure tube was closed and stirred at 85 ºC for 24 hours in an oil bath. Once the reaction finished, it was extracted with EtOAc (3x10 mL), the organic phase dried over $MgSO_4$ and the solvent evaporated under reduced pressure (15 Torr). The residue was purified by column chromatography using a gradient of hexane/EtOAc (starting with hexane, then hexane/EtOAc 99:1, 98:2, 97:3…) until complete elution of the desired compound. The product was obtained as a slightly soluble yellow solid with 73% yield (calculated with >99% *N,N'*-diphenylformamide as internal standard via 400 MHz ^{1}H-NMR).

Yellow solid; R_f: 0.21 (hexane/EtOAc 99:1); ^{1}H-NMR (400 MHz, $CDCl_3$) δ= 8.21 (d, *J* = 0.9 Hz, 1H), 8.02 (d, *J* = 1.9 Hz, 1H), 7.94 (d, *J* = 8.5 Hz, 1H), 7.84 – 7.80 (m, 5H), 7.73 (d, *J* = 8.5 Hz, 1H), 7.70 (d, *J* = 8.7 Hz, 1H), 7.57 – 7.51 (m, 2H), 7.49 – 7.44 (m, 3H) ppm; LRMS (EI, DIP): *m/z* (%) 419.0 (M^++3, 1), 418.0 (M^++2, 6), 417.0 (M^++1, 21), 416.1 (M^+, 75), 415.1 (M^+-1, 35), 414.1 (M^+-2, 76), 336.1 (16), 335.1 (54), 334.1 (100), 333.2 (22), 332.1 (25), 300.1 (14), 167.5 (38), 166.3 (14); HRMS (EI, QTOF): *m/z* found for $C_{24}H_{15}BrS$ 416.0061, calculated 416.00778 (M^+); IR (neat) ν_{max} 1616, 1583, 1500, 1319, 1059, 960, 910, 883, 831, 713, 633 cm^{-1}.

1.3.7. 14-bromonaphtho[1',2':5,6]phenanthro[4,3-*b*]thiophene

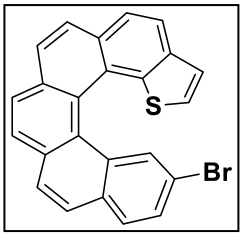

This compound was prepared using our photochemical setup described above. (*E*)-8-(2-(7-bromonaphthalen-2-yl)vinyl)naphtho[1,2-*b*]thiophene (12.46 mg, 0.03 mmol, 1 eq.), iodine (11.42 mg, 0.045 mmol, 1.5 eq.) and 1,2-epoxybutane (0.261 mL, 3 mmol, 100 eq.) were mixed with 200 mL of benzene in a Schlenk tube equipped with a vertical condenser and a gas trap on the top. Then, argon was bubbled into the tube for 10 minutes. The reaction was stirred and irradiated with 2x50 W (365 nm) LEDs for 7 hours. Once the reaction was completed, it

was washed with aqueous $NaHSO_3$ (15 mL, 1 M) and three times with water (3x15 mL). The organic phase was dried over $MgSO_4$ and the solvent evaporated and recovered under reduced pressure (15 Torr). The crude of the reaction was purified by column chromatography using a gradient of hexane/DCM (starting with hexane, then hexane/DCM 99:1, 98:2, 97:3…) until complete elution of the desired compound. A yellow solid was obtained as the product with 53% yield (calculated with >99% *N,N'*-diphenylformamide as internal standard via 400 MHz ^{1}H-NMR).

Yellow solid; R_f: 0.43 (hexane/DCM 7:3); ^{1}H-NMR (400 MHz, $CDCl_3$) δ= 8.11 (d, J = 1.9 Hz, 1H), 8.08 (d, J = 8.4 Hz, 1H), 8.05 (d, J = 8.4 Hz, 1H), 8.01 – 7.96 (m, 4H), 7.94 (d, J = 8.6 Hz, 1H), 7.91 (d, J = 8.4 Hz, 1H), 7.81 (d, J = 8.5 Hz, 1H), 7.46 (dd, J = 8.5, 1.9 Hz, 1H), 7.32 (d, J = 5.4 Hz, 1H), 7.14 (d, J = 5.4 Hz, 1H) ppm; ^{13}C-NMR (101 MHz, $CDCl_3$) δ= 138.66 (-C-, 1C), 137.03 (-C-, 1C), 133.15 (-C-, 1C), 132.21 (-C-, 1C), 131.94 (-C, 1C), 131.01 (-C-, 1C), 130.38 (-C-, 1C), 129.58 (-CH-, 1C), 129.37 (-CH-, 1C), 129.15 (-CH-, 1C), 128.52 (-CH-, 1C), 127.51 (-CH-, 1C), 127.38 (-CH-, 1C), 127.31 (-CH-, 1C), 127.08 (-C-, 1C), 127.06 (-CH-, 1C), 125.84 (-CH-, 1C), 125.36 (-CH-, 1C), 125.16 (-C-, 1C), 125.13 (-CH-, 1C), 124.00 (-C-, 1C), 123.28 (-CH-, 1C), 123.18 (-CH-, 1C), 119.62 (-C-, 1C) ppm; LRMS (EI, DIP) *m/z* (%) 416.0 (M^++2, 0.5), 415.0 (M^++1, 2), 414.0 (M^+, 26), 413.0 (M^+-1, 7), 412.0 (M^+-2, 25), 333.1 (10), 332.0 (25), 301.1 (27), 300.1 (100), 299.1 (12), 298.1 (16), 287.1 (17), 166.1 (19), 165.1 (11), 150.0 (46), 149.1 (36), 143.5 (14), 111.1 (16), 109.1 (12), 97.1 (21), 95.1 (17), 85.1 (16), 83.1 (20), 81.1 (16), 71.1 (24), 69.1 (24), 67.1 (11), 57.1 (37), 55.1 (26), 43.1 (34), 41.1 (16); HRMS (EI, QTOF) *m/z* found for $C_{14}H_{13}BrS$ 413.9893; calculated 413.99213 (M^+); IR (neat) ν_{max} 2924, 2854, 1589, 1485, 1421, 1286, 1082, 891, 843, 804, 725, 700 cm^{-1}.

1.3.8. (*E*)-8-(2-(naphto[2,1-*b*]thiophen-8-yl)vinyl)naphto[1,2-*b*]thiophene

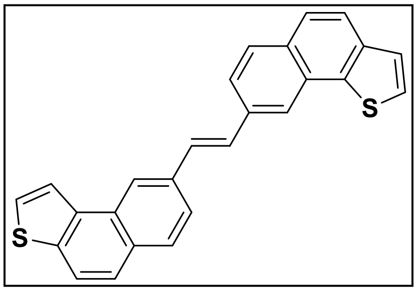

In a pressure tube, $PdCl_2$ (10.64 mg, 0.06 mmol, 0,1 eq.), PPh_3 (31.47 mg, 0.12 mmol, 0,2 eq.), Cs_2CO_3 (586.48 mg, 1.8 mmol, 3 eq.), 8-bromonaphtho[2,1-*b*]thiophene (157.89 mg, 0.6 mmol, 1 eq.) and (*E*)-4,4,5,5-tetramethyl-2-(2-(naphtho[1,2-*b*]thiophen-8-yl)vinyl)-1,3,2-dioxaborolane (302.63 mg, 0.9 mmol, 1.5 eq.) were added. The tube was sealed with a septum and three cycles of vacuum/argon were performed to reach inert conditions. Then, THF (3.6 mL) and H_2O (0.4 mL) were added with purged syringes. The pressure tube was closed and the reaction was stirred at 100 ºC for 20 hours in an oil bath. Once the reaction was completed, it was filtered with a Büchner funnel and washed with EtOAc and H_2O. The product was obtained (164.86 mg, 0.42 mmol, 70% yield) as a greenish powder.

Greenish powder; LRMS (EI, DIP): *m/z* (%) 395.1 (M^++3, 3), 394.1 (M^++2, 16), 393.1 (M^++1, 39), 392.1 (M^+, 100), 391.2 (43), 390.2 (22), 358.1 (12), 196.0 (10); HRMS (EI, QTOF): *m/z* found for $C_{26}H_{16}S_2$ 392.0674, calculated 392.0693376 (M^+); IR (neat) ν_{max} 1618, 1321, 1263, 1190, 1151, 1086, 960, 877, 829, 791, 775, 710, 656 cm^{-1}.

1.3.9. (*E*)-14-(2-(naphtho[2,1-*b*]thiophen-8-yl)vinyl)naphtho[1',2':5,6]phenanthro[4,3-*b*]thiophene

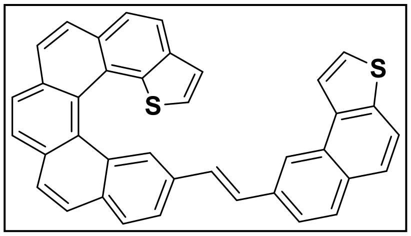

In a pressure tube, $PdCl_2$ (1.77 mg, 0.01 mmol, 0.1 eq.), PPh_3 (2.62 mg, 0.02 mmol, 0.2 eq.), Cs_2CO_3 (97.75 mg, 0.3 mmol, 3 eq.), 14-bromonaphtho[1',2':5,6]phenanthro[4,3-*b*]thiophene (41.33 mg, 0.1 mmol, 1 eq.), (*E*)-4,4,5,5-tetramethyl-2-(2-(naphtho[2,1-*b*]thiophen-8-yl)vinyl)-1,3,2-dioxaborolane (50.44 mg, 0.15 mmol, 1.5 eq.) were added. The tube was sealed with a septum and three cycles of vacuum/argon were performed. Then, THF (0.6 mL) and H_2O (0.067 mL) were added with purged syringes. The reaction was stirred for 24 hours at 100 ºC. Once the reaction time has elapsed, the mixture was extracted with EtOAc (3x10 mL), the organic phase was dried over $MgSO_4$ and evaporated under reduced pressure (15 Torr). The crude of the reaction was purified by column chromatography using a gradient of hexane/DCM (starting with hexane, then hexane/DCM 99:1, 98:2, 97:3…) until complete elution of the desired compound. The product was obtained as a slightly soluble yellow solid with 77% yield (calculated with >99% *N,N'*-diphenylformamide as internal standard via 400 MHz ^{1}H-NMR).

Yellow solid; R_f: 0.14 (hexane/DCM 9:1); ^{1}H-NMR (400 MHz, $CDCl_3$) δ= 8.26 (d, *J* = 1.2 Hz, 1H), 8.16 (d, *J* = 8.4 Hz, 1H), 8.14 – 8.09 (m, 3H), 8.03 (d, *J* = 5.4 Hz, 1H), 8.00 (dd, *J* = 7.2, 1.3 Hz, 4H), 7.96 (dd, *J* = 8.3, 4.4 Hz, 2H), 7.84 (d, *J* = 8.7 Hz, 2H), 7.68 (d, *J* = 8.8 Hz, 1H), 7.65 (d, *J* = 5.5 Hz, 1H), 7.60 (dd, *J* = 8.3, 1.6 Hz, 1H), 7.54 (dd, *J* = 8.5, 1.6 Hz, 1H), 7.29 (d, *J* = 5.4 Hz, 1H), 7.12 (d, *J* = 5.4 Hz, 1H), 6.90 (d, *J* = 16.2 Hz, 1H), 6.56 (d, *J* = 16.2 Hz, 1H) ppm; LRMS (EI, DIP) *m/z* (%) 546.1 (M^++4, 0.8), 545.1 (M^++3, 3), 544.1 (M^++2, 11), 543.1 (M^++1, 26), 542.1 (M^+, 59), 325.1 (14), 324.1 (13), 319.0 (10), 314.1 (21), 313.1 (36), 300.1 (100), 298.1 (12), 287.1 (10), 271.1 (17), 241.0 (11), 197.0 (16), 44.1 (65), 28.1 (11); HRMS (EI, QTOF) *m/z* found for $C_{38}H_{22}S_2$ 542.1156; calculated 542.1162852 (M^+); IR (neat) ν_{max} 2622, 2854, 1464, 1375, 1267, 957, 906, 883, 839, 725, 710, 673, 640 cm^{-1}.

1.3.10. 2,7-bis((E)-2-(naphtho[2,1-b]tiophen-8-yl)vinyl)naphthalene

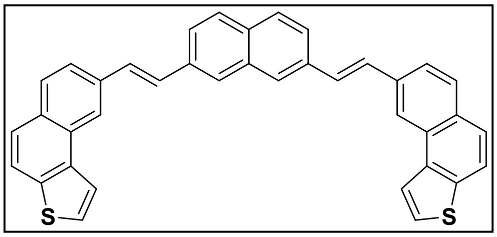

This compound was prepared by adapting a Suzuki coupling described in the literature to our substrate.[7] In a pressure tube provide with a stirbar, naphthalene-2,7-diyl bis(trifluoromethanesulfonate) (0.364 g, 0.85 mmol, 1 eq.), (E)-4,4,5,5-tetramethyl-2-(2-(naphtho[2,1-b]tiophen-8-yl)vinyl)-1,3,2-dioxoborolane (0.645 g, 1.9 mmol, 2.2 eq.) and Cs_2CO_3 (3.36 g, 10.2 mmol, 12 eq.) were added. The tube was sealed with the septum and after three cycles of vacuum/argon, 29 mL of dry DMF was added by syringe. Resulted solution was degassed by three cycles of freeze-pump-thaw. Then tetrakis(triphenylphosphine)palladium(0) (0.196 g, 0.17 mmol, 0.2 eq.) was added in one portion. After that the tube was closed and heated in an oil bath at 80 ℃, and kept at this temperature overnight. After cooling down to room temperature, the formed precipitate was filtered out and washed with water (3 x 5 mL) and CH_2Cl_2 (3 x 10 mL) to afford the product as greyish-green powder in 90% yield.

[7] Dadvand, A.; Moiseev, A. G.; Sawabe, K.; Sun, W. H.; Djukic, B.; Chung, I.; Takenobu, T.; Rosei, F.; Perepichka, D. F. (2012) Maximizing Field-Effect Mobility and Solid-State Luminescence in Organic Semiconductors Angew. Chem. Int. Ed. 51, 3837-3841. DOI: 10.1002/anie.201108184

Greyish-green solid; MS (EI, DIP) *m/z* 545.90 (M^++2, 40.28), 544.90 (M^++1, 46.58), 543.90 (M^+, 100), 357.90 (18.45), 333.90 (15.05), 271.20 (24.65), 254.00 (14.82). IR (neat) ν_{max}: 1620, 1512, 1369, 1196, 1153, 1088, 957, 887, 837, 779, 710, 671 cm^{-1}.

1.3.11. **2,11-bis((E)-2-(benzo[b]tiophen-6-yl)vinyl)benzo[c]phenanthrene**

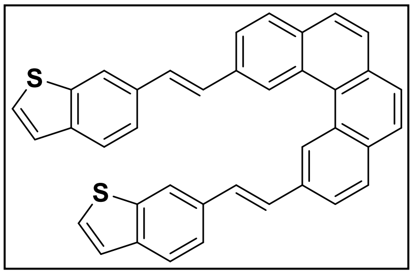

The synthesis of this compound was revived following the conditions of the Suzuki reaction used by our group for the synthesis of others bis-stilbenic precursors. In an over-dried pressure tube $PdCl_2$ (17.33 mg; 0.1 mmol; 0.20 eq.), PPh_3 (52.46 mg; 0.2 mmol; 0.40 eq.), Cs_2CO_3 (977.46 mg; 3 mmol; 6 eq.), 2,11-dibromobenzo[c]phenanthrene (193.10 mg; 0.5 mmol; 1 eq.) were added. The tube was sealed with a septum and after three cycles of vacuum/argon, (E)-2-(2-benzo[b]thiophen-6-yl)vinyl)-4,4,5,5-tetramethyl-1,3,2-dioxaborolane (429.30 mg, 1.5 mmol, 3 eq.) was added dissolved in 3.6 mL of THF followed by 0.4 mL of H_2O, both of them added via syringe. After that the tube was closed and heated in an oil bath at 85℃ for 20 hours. An insoluble solid in suspension was observed in the tube. The insoluble, greyish-green solid in 60% yield, was filtered and washed with H_2O (3 x 5 mL) and CH_2Cl_2 (3 x 10 mL).

Greyish-green solid; MS (EI, DIP) *m/z* 546.2 (M^++2, 19.56), 545.2 (M^++1, 47.95), 544.2 (M^+, 100), 408.1 (7.18), 395.1 (11.12), 382.1 (10.65), 272.1 (16.74), 204.8 (6.01). IR (neat) νmax: 2974, 2889, 1597, 1454, 1392, 1084, 1045, 953, 879, 841, 814, 752, 694 cm^{-1}.

1.3.12. **3,6-bis((E)-2-(naphtho[2,1-b]tiophen-8-yl)vinyl)phenanthrene**

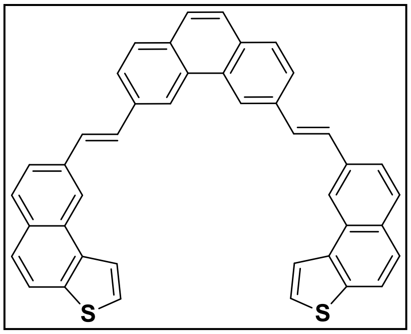

The synthesis of this compound was revived following the conditions of the Suzuki reaction used by our group for the synthesis of others bis-stilbenic precursors. In an over-dried pressure tube $PdCl_2$ (8.86 mg; 0.05 mmol; 0.20 eq.), PPh_3 (26.22 mg; 0.1 mmol; 0.40 eq.), Cs_2CO_3 (488.73 mg; 1.5 mmol; 6 eq.), 3,6-dibromophenanthrene (84.10 mg; 0.25 mmol; 1 eq.) were added. The tube was sealed with a septum and after three cycles of vacuum/argon, (E)-4,4,5,5-tetramethyl-2-(2-(naphtho[2,1-b]tiophen-8-yl)vinyl)-1,3,2-dioxoborolane (252.11 mg, 0.75 mmol, 3 eq.) was added dissolved in 1.8 mL of THF followed by 0.2 mL of H_2O, both of them added via syringe. After that the tube was closed and heated in an oil bath at 85℃ for 20 hours. An insoluble solid in suspension was observed in the tube. The insoluble solid, a yellow solid in 90% yield, was filtered and cleaned with H_2O (3 x 5 mL) and CH_2Cl_2 (3 x 10 mL).

Yellow solid; MS (EI, DIP) *m/z*: 596.1 (M^++2, 19.56), 595.1 (M^++1, 47.95), 594.1 (M^+, 100), 545.1 (28.31), 544.1 (70.71), 408.1 (12.65), 358.1 (13.74), 297.1 (20.01), 272.0 (15.02), 197.0 (10.81). IR (neat) νmax: 3062, 3020, 1616, 1373, 1192, 1153, 953, 875, 833, 710, 663 cm^{-1}.

1.3.13. **3,6-bis((E)-2-(naphtho[1,2-b]tiophen-8-yl)vinyl)phenanthrene**

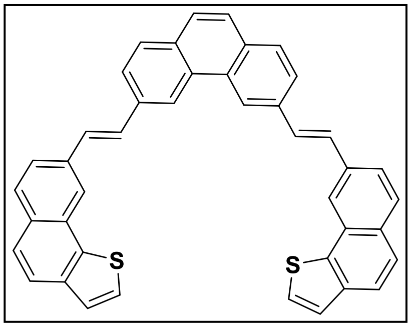

This compound was prepared following the previous procedure, but as starting reagents were used (E)-4,4,5,5-tetramethyl-2-(2-(naphtho[1,2-b]tiophen-8-yl)vinyl)-1,3,2-dioxoborolane. The insoluble solid, a yellow solid in 90% yield, was filtered and cleaned with H_2O (3 x 5 mL) and CH_2Cl_2 (3 x 10 mL).

Yellow solid; MS (EI, DIP) *m/z* 596.1 (M^++2, 19.85), 595.1 (M^++1, 46.77), 594.1 (M^+, 100), 546.1 (15.95), 545.1 (30.01), 544.1 (69.91), 408.1 (13.04), 384.1 (8.23), 358.1 (12.57), 297.1 (25.66), 272.0 (17.10), 197.0 (13.71). IR (neat) νmax: 2974, 2885, 1612, 1312, 1261, 1088, 1045, 949, 875, 833, 702 cm^{-1}.

General photocyclization procedure: In an oven-dried 250 mL Schlenk tube, the corresponding precursors for each helicenes (16.34 mg, 0.03 mmol, 1 eq.) were added, followed by 200 mL of benzene. The mixture was stirred and heated a little bit with a heat gun to partially dissolve the reagent. After that the iodine (22.84 mg, 0.09 mmol, 3 eq.) and 1,2-epoxybutane (0.3 mL, 3 mmol, 100 eq.) were added. The tube was capped with a silicone bubbler and the solution was bubbled with Ar for 15 minutes. Then the 2 LEDs boards (50 W each) of the photochemical setup were turned on and the Ar bubbling was continued for 15 additional minutes. The mixture was irradiated with LED UV light overnight (12 hours). After the reaction was completed, the crude was washed with aqueous NaHSO3, dried over magnesium sulphate, filtered, and the solvent evaporated under reduced pressure (15 Torr).

1.3.14. **1,14-dithia[7]helicene or *exo-endo*-dithia[7]helicene**

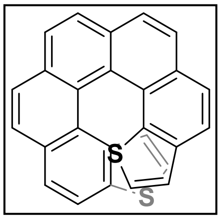

This compound was prepared following the previous photocyclization procedure and as starting reagents (*E*)-8-(2-(naphto[2,1-*b*]thiophen-8-yl)vinyl)naphto[1,2-*b*]thiophene was used. The crude was purified by column chromatography on silica gel using a gradient of hexane/DCM (starting with hexane, then hexane/DCM 99:1, 98:2, 97:3…). The product was obtained as a yellowish solid with 69% yield (calculated with >99% *N,N'*-diphenylformamide as internal standard via 400 MHz ^{1}H-NMR).

Yellowish solid; R_f: 0.36 (hexane/DCM 8:2); ^{1}H-NMR (400 MHz, $CDCl_3$) δ= 8.14 (d, *J* = 8.3 Hz, 1H), 8.10 (d, *J* = 8.4 Hz, 1H), 8.07 (d, *J* = 8.1 Hz, 1H), 8.05 (d, *J* = 8.1 Hz, 1H), 8.04 (d, *J* = 8.4 Hz, 1H), 7.98 (d, *J* = 8.4 Hz, 1H), 7.96 (d, *J* = 8.5 Hz, 1H), 7.94 (d, *J* = 8.4 Hz, 1H), 7.93 (d, *J* = 8.4 Hz, 1H), 7.84 (d, *J* = 8.3 Hz, 1H), 7.02 (d, *J* = 5.4 Hz, 1H), 6.80 (d, *J* = 5.4 Hz, 1H), 6.63 (d, *J* = 5.6 Hz, 2H), 6.33 (d, *J* = 5.6 Hz, 1H) ppm; ^{13}C-NMR (101 MHz, $CDCl_3$) δ= 138.57 (-C-, 1C), 138.18, (-C-, 1C) 136.47 (-C-, 1C), 135.77 (-C-, 1C), 132.57 (-C-, 1C), 132.36 (-C-, 1C), 130.63 (-C-, 1C), 129.95 (-C-, 1C), 128.48 (-CH-, 1C), 128.27 (-CH-, 1C), 128.24 (-C-, 1C), 127.75 (-C-, 1C), 127.07 (-CH-, 1C), 126.82 (-CH-, 1C), 125.72 (-CH-, 1C), 125.30 (-CH-, 1C), 124.98 (-CH-, 1C), 124.85 (-CH-, 1C), 124.68 (-C-, 1C), 124.46 (-CH-, 1C), 124.10 (-CH-, 1C), 123.51 (-C-, 1C), 123.21 (-CH-, 1C), 122.62 (-CH-, 1C), 122.34 (-CH-, 1C), 121.67 (-CH-, 1C) ppm; LRMS (EI, DIP) *m/z* (%) 393.0 (M^++3, 3), 392.0 (M^++2, 13), 391.0 (M^++1, 30), 390.0 (M^+, 100), 357.1 (27), 356.0 (46), 345.0 (19), 344.0 (17), 343.0 (31), 300.1 (12), 195.0 (11), 194.1 (14), 178.1 (28), 171.5 (17), 44.1 (12); HRMS (EI, QTOF) *m/z* found for $C_{26}H_{14}S_2$ 390.0529; calculated 390.0536884 (M^+); IR (neat) ν_{max} 3043, 2922, 2852, 1321, 1273, 1194, 1149, 1117, 841, 316, 741, 696, 66, 623, 611 cm^{-1}.

1.3.15. **1,20-dithia[10]helicene or *exo-endo*-dithia[10]helicene**

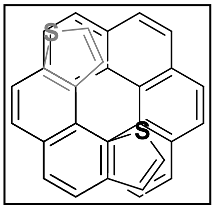

This compound was prepared following the previous photocyclization procedure and as starting reagents (*E*)-14-(2-(naphtho[2,1-*b*]thiophen-8-yl)vinyl)naphtho[1',2':5,6]phenanthro[4,3-*b*]thiophene was used. The crude of the reaction was purified by column chromatography using a gradient of hexane/DCM (starting with hexane, then hexane/DCM 99:1, 98:2, 97:3…) until complete elution of the desired compound. The product was crystallized in toluene or CH_2Cl_2 to obtain clean yellow crystals.

Yellow crystals in 33% yield; R_f: 0.19 (hexane/DCM 9:1); ^{1}H-NMR (400 MHz, $CDCl_3$) δ= 8.02 (dd, *J* = 8.2, 2.6 Hz, 2H), 7.78 (d, *J* = 8.2 Hz, 2H), 7.53 (dd, *J* = 8.5, 0.7 Hz, 1H), 7.47 (d, *J* = 8.4 Hz, 1H), 7.42 (dd, *J* = 8.2, 2.3 Hz, 2H), 7.32-7.27 (m, 4H), 7.16 (d, *J* = 8.2 Hz, 1H), 7.15 (d, *J* = 8.2 Hz, 1H), 7.08 (dd, *J* = 8.4, 1.3 Hz, 2H), 6.86 (d, *J* = 5.4 Hz, 1H), 6.68 (d, *J* = 5.4 Hz, 1H), 6.46 (d, *J* = 5.6 Hz, 1H), 6.03 (d, *J* = 5.6, 0,8 Hz, 1H) ppm; LRMS (EI, DIP) *m/z* (%) 544.1 (M^++4, 2), 543.1 (M^++3, 6), 542.1 (M^++2, 18), 541.1 (M^++1, 43), 540.1 (M^+, 100), 506.1 (10), 270.0 (18), 269.0 (20), 268.1 (19), 253.0 (20), 252.1 (19), 245.7 (17); HRMS (EI, QTOF) *m/z* found for $C_{38}H_{20}S_2$ 540.0997; calculated 540.100636 (M^+); IR (neat) ν_{max} 2922, 2854, 1728, 1464, 1269, 1120, 1076, 843, 802, 702 cm^{-1}.

1.3.16. **3,20-Dithia[10]helicene or *exo*-dithia[10]helicene**

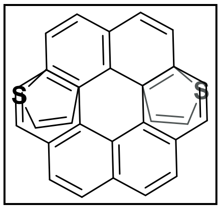

This compound was prepared following the previous photocyclization procedure and as starting reagents 2,7-*bis*((*E*)-2-naphtho[2,1-*b*]tiophen-8-yl)vinyl)naphthalene was used. The residue was purified by column chromatography on silica gel (hexane-CH_2Cl_2 6:4) to obtain a yellow solid. The product was crystallized in benzene or CH_2Cl_2 to obtain clean yellow crystals.

Yellow crystals in 40% yield; R_f= 0.6 (hexane-CH_2Cl_2 6:4); ^{1}H-NMR ($CDCl_3$, 400 MHz): δ= 7.99 (d, *J* = 8.3 Hz, 2H), 7.73 (d, *J* = 8.2 Hz, 2H), 7.53 (dd, *J* = 8.5, 0.8 Hz, 2H) 7.41 (d, *J* = 8.1 Hz, 2H), 7.31 (2d, *J* = 8.4 Hz, 4H), 7.20 (d, *J* = 8.1 Hz, 2H), 7.12 (d, *J* = 8.3 Hz, 2H), 6.48 (dd, *J* = 5.5, 0.5 Hz, 2H), 6.05 (dd, *J* = 5.5, 0.8 Hz, 2H) ppm. ^{13}C-NMR ($CDCl_3$, 101 MHz): δ= 133.66 (-C-, 2C), 132.39 (-C-, 1C), 131.00 (-C-, 2C), 129.99 (-C-, 2C), 128.38 (-C-, 2C), 127.30 (-CH-, 1C), 126.62 (-CH-, 2C), 126.54 (-CH-, 2C), 126.45 (-CH-, 2C), 125.71 (-CH-, 2C), 125.67 (-C-, 2C), 124.98 (-CH-, 2C), 124.96 (-CH-, 2C), 124.70 (-C-, 2C), 124.58 (-C-, 2C), 122.80 (-CH-, 2C), 122.69 (-C-, 2C), 121.82 (-CH-, 2C), 119.92 (-CH-, 2C) ppm. MS (EI, DIP) *m/z*: 542.1 (M^++2, 18.00), 541.2 (M^++1, 43.41), 540.2 (M^+, 100), 270.0 (13.26), 252.2 (18.51), 245.6 (10.89). HRMS (EI, QTOF) *m/z* found for $C_{38}H_{20}S_2$ 540.0994; calculated 540.100636 (M^+). IR (neat) ν_{max}: 2974, 1454, 1404, 1304, 1192, 1088, 1045, 883, 725, 690 cm^{-1}.

1.3.17. **1,22-Dithia[10]helicene or *endo*-dithia[10]helicene**

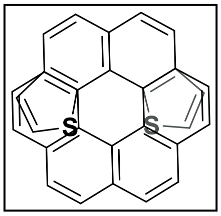

This compound was prepared following the previous photocyclization procedure and as starting reagents 2,11-*bis*((*E*)-2-(benzo[*b*]tiophen-6-yl)vinyl)benzo[*c*]phenanthrene was used. The residue was purified by column chromatography on silica gel using different polarity mixture first (hexane-CH_2Cl_2 8:2) to eliminate some impurities and then (hexane-CH_2Cl_2 6:4) to obtain a yellow solid. The product was crystallized in toluene to obtain clean yellow crystals.

Yellow crystals in 58% yield; R_f= 0.5 (hexane-CH_2Cl_2 6:4); ^{1}H-NMR ($CDCl_3$, 300 MHz): δ= 8.07 (d, *J* = 8.2 Hz, 2H), 7.85 (d, *J* = 8.2 Hz, 2H), 7.50 (d, *J* = 8.4 Hz, 2H), 7.45 (d, *J* = 8.2 Hz, 2H), 7.34 (d, *J*

= 8.5 Hz, 2H), 7.31 (d, *J* = 8.4 Hz, 2H), 7.14 (d, *J* = 8.2 Hz, 2H), 7.06 (d, *J* = 8.4 Hz, 2H), 6.87 (d, *J* = 5.4 Hz, 2H), 6.69 (d, *J* = 5.4 Hz, 2H) ppm. ^{13}C-NMR ($CDCl_3$, 101 MHz): 137.71 (-C-, 2C), 135.06 (-C-, 2C), 132.78 (-C-, 1C), 132.65 (-C-, 2C), 131.09 (-C-, 2C), 130.20 (-C-, 2C), 127.88 (-CH-, 1C), 127.25 (-CH-, 2C), 127.18 (-CH-, 2C), 126.13 (-CH-, 2C), 125.71 (-CH-, 2C), 125.30 (-CH-, 2C), 124.87 (-CH-, 2C), 124.63 (-C-, 2C), 124.46 (-C-, 2C), 124.40 (-C-, 2C), 123.33 (-CH-, 2C), 121.78 (-CH-, 2C), 121.15 (-CH-, 2C) ppm. MS (EI, DIP) *m/z*: 542.1 (M^++2, 17.41), 541.1 (M^++1, 42.57), 540.1 (M^+, 100), 493.1 (7.51), 270.1 (18.70), 252.2 (8.88), 229.6 (4.71). HRMS (EI, QTOF) *m/z* found for $C_{38}H_{20}S_2$ 540.0999; calculated 540.100636 (M^+). IR (neat) ν_{max}: 3039, 1277, 1142, 1088, 949, 837, 725, 686 cm^{-1}.

1.3.18. **3,22-Dithia[11]helicene or *exo*-dithia[11]helicene**

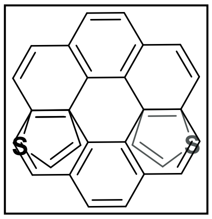

This compound was prepared following the previous photocyclization procedure, and as starting reagents 3,6-*bis*((*E*)-2-(naphtho[2,1- *b*]tiophen-8-yl)vinyl)phenanthrene was used. The residue was purified by column chromatography on silica gel (hexane-CH_2Cl_2 6:4) to obtain a yellow solid. The product was crystallized in toluene to obtain yellow crystals.

Yellow crystals in 50% yield; R_f= 0.45 (hexane-CH_2Cl_2 6:4); ^{1}H-NMR ($CDCl_3$, 400 MHz): δ= 7.61 (s, 2H), 7.47 (dd, *J*= 8.5, 0.8 Hz, 2H), 7.38 (d, *J*= 8.2 Hz, 2H), 7.34 (d, *J*= 8.4 Hz, 2H), 7.27 (d, *J*= 8.3 Hz, 2H), 7.23 (d, *J*= 8.3 Hz, 2H), 7.22 (d, *J*= 8.2 Hz, 2H), 7.22 (s, 4H), 6.37 (dd, *J*= 5.6, 0.4 Hz, 2H), 5.81 (dd, *J*= 5.6, 0.8 Hz, 2H) ppm. ^{13}C-NMR ($CDCl_3$, 101 MHz): δ= 137.44 (-C-, 2C), 133.97 (-C-, 2C), 131.95 (-C-, 2C), 131.10 (-CH-, 2C), 130.04 (-C-, 2C), 127.24 (-C-, 2C), 126.76 (-CH-, 2H), 126.46 (-CH-, 2C), 126.18 (-CH-, 2C), 126.14 (-CH-, 2C), 125.63 (-CH-, 2C), 125.54 (-CH-, 2C), 124.62 (-C-, 2C), 124.57 (-C-, 2C), 124.49 (-C-, 2C), 123.51 (-CH-, 2C), 122.72 (-CH-, 2C), 121.59 (-CH-, 2C), 121.08 (-C-, 2C), 119.85 (-CH-, 2C), 118.21 (-C-, 2C) ppm. MS (EI, DIP) *m/z*: 592.2 (M^++2, 19.93), 591.2 (M^++1, 48.52), 590.2 (M^+, 100), 306.1 (11.78), 295.1 (25.66), 282.1 (11.07), 277.0 (18.16), 276.1 (15.06), 263.1 (10.43). HRMS (EI, QTOF) *m/z* found for $C_{42}H_{22}S_2$ 590.1156; calculated 590.1162852 (M^+). IR (neat) ν_{max}: 3039, 1315, 1292, 1138, 1092, 949, 891, 833, 783, 714, 683 cm^{-1}.

1.3.19. **1,24-Dithia[11]helicene or *endo*-dithia[11]helicene**

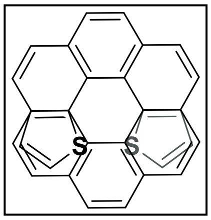

This compound was prepared following the previous photocyclization procedure, and as starting reagents 3,6-*bis*((*E*)-2-(naphtho[1,2-*b*]tiophen-8-yl)vinyl)phenanthrene was used. The residue was purified by column chromatography on silica gel (hexane-CH_2Cl_2 6:4) to obtain a yellow solid. The product was crystallized in CH_2Cl_2 or toluene to obtain yellow crystals.

Yellow crystals in 46% yield; R_f= 0.52 (hexane-CH_2Cl_2 6:4); ^{1}H-NMR ($CDCl_3$, 300 MHz): δ= 7.72 (s, 2H), 7.46 (d, *J*= 8.2 Hz, 2H), 7.44 (d, *J*= 8.4 Hz, 2H), 7.39 (d, *J*= 8.4 Hz, 2H), 7.30 (d, *J*=8.4 Hz, 2H), 7.24 (d, *J*= 8.2 Hz, 2H), 7.23 (d, *J*= 8.3 Hz, 2H), 7.21 (s, 4H), 6.83 (d, *J*= 5.4 Hz, 2H), 6.63 (d, *J*= 5.4 Hz, 2H) ppm. ^{13}C-NMR ($CDCl_3$, 101 MHz): δ= 137.20 (-C-, 2C), 135.32 (-C-, 2C), 132.86 (-C-, 2C), 131.39 (-C-, 2C), 130.88 (-C-, 2C), 130.05 (-C-, 2C), 127.33 (-C-, 2C), 127.03 (-CH-, 2C), 127.00 (-CH-, 2C), 126.29 (-CH-, 2C), 126.19 (-CH-, 2C), 125.92 (-CH-, 2C), 125.69 (-CH-, 2C), 125.61 (-CH-, 2C), 124.69 (-CH-, 2C), 124.37 (-C-, 2C), 123.12 (-CH-, 2C), 122.73 (-C-, 2C), 122.72 (-C-, 2C), 121.56 (-CH-, 2C), 120.92 (-CH-, 2C) ppm. MS (EI, DIP) *m/z*: 592.2 (M^++2, 19.12), 591.1 (M^++1, 44.89), 590.2 (M^+, 100), 295.1 (22.87), 277.2 (10.76), 270.7 (12.09), 245.1 (6.92). HRMS (EI, QTOF) *m/z* found for $C_{42}H_{22}S_2$ 590.1160; calculated 590.1162852 (M^+). IR (neat) ν_{max}: 3039, 1300, 1265, 949, 833, 752, 717, 679 cm^{-1}.

1.4. ^{1}H-NMR and ^{13}C-NMR spectra of compounds

1.4.1. **Naphthalene-2,7-diyl bis(trifluoromethanesulfonate) (300 Mhz, $CDCl_3$)**

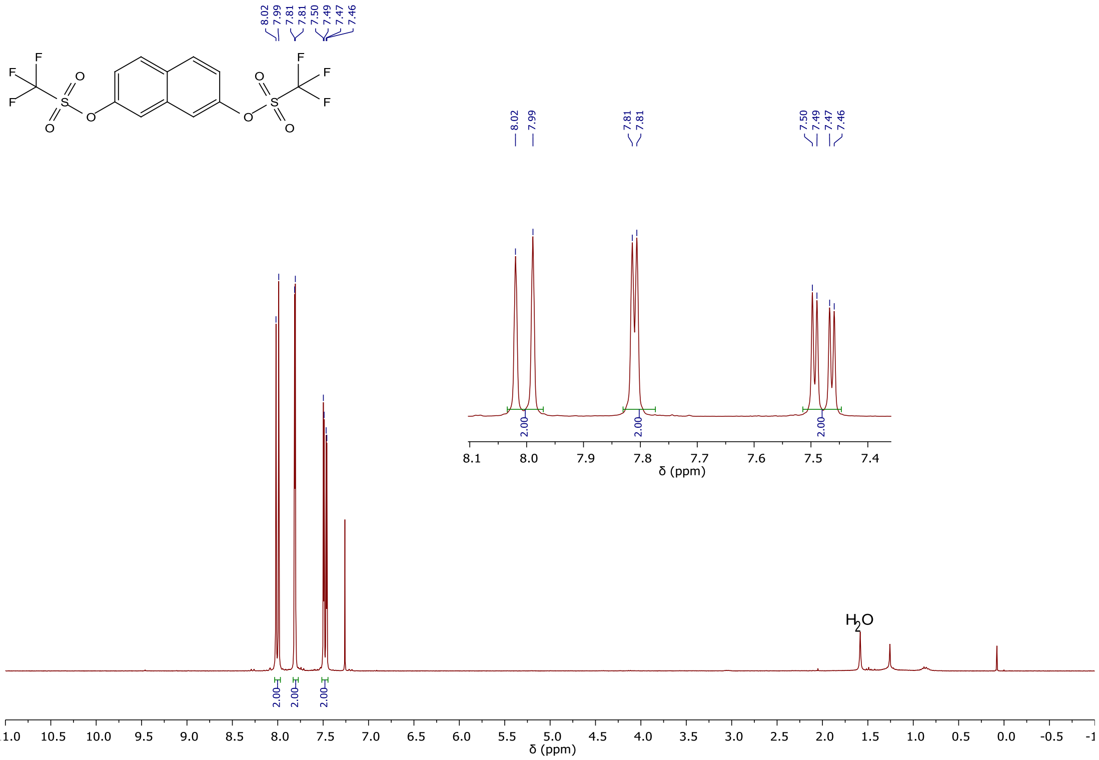

Naphthalene-2,7-diyl bis(trifluoromethanesulfonate) (75 Mhz, $CDCl_3$)

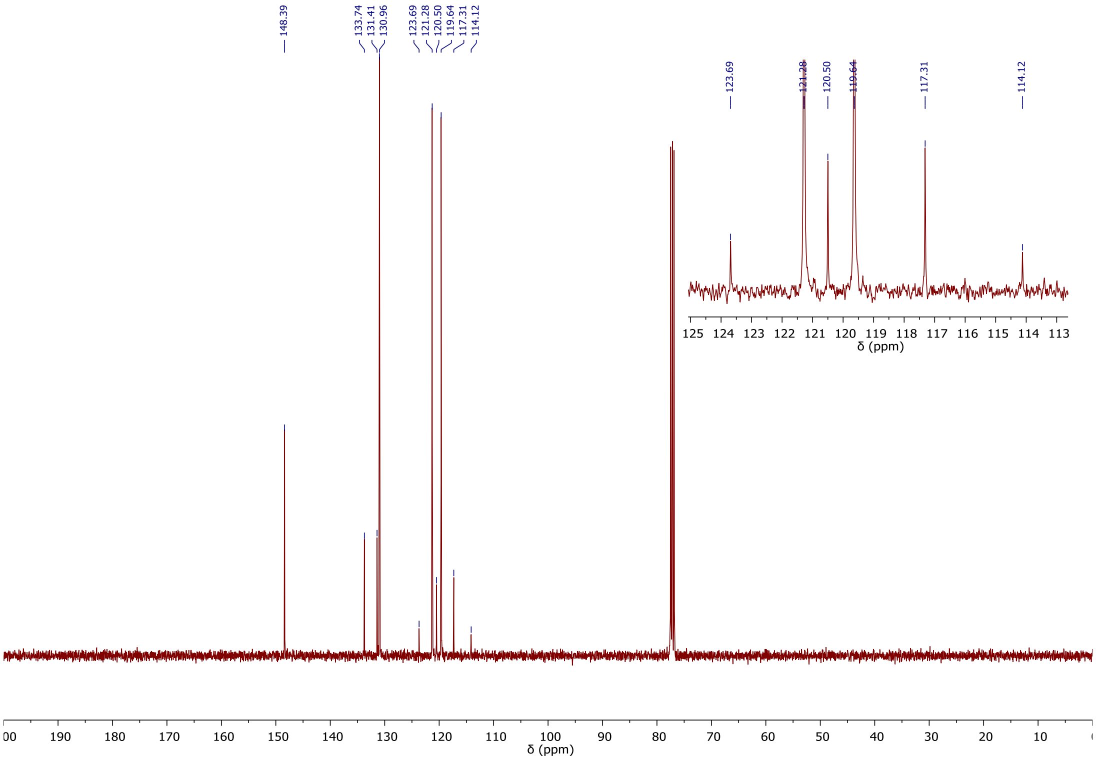

1.4.2. **(*E*)-2-(4-Bromostyryl)-4,4,5,5-tetramethyl-1,3,2-dioxaborolane (300 MHz, $CDCl_3$)**

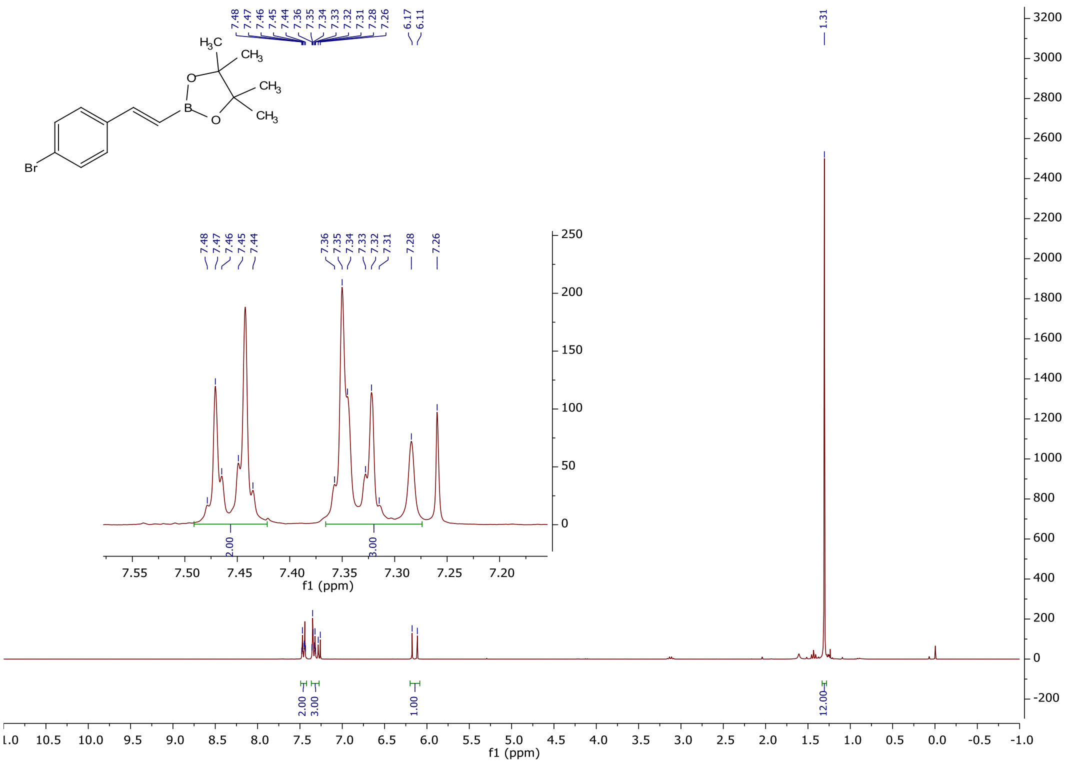

(*E*)-2-(4-Bromostyryl)-4,4,5,5-tetramethyl-1,3,2-dioxaborolane (75 MHz, $CDCl_3$)

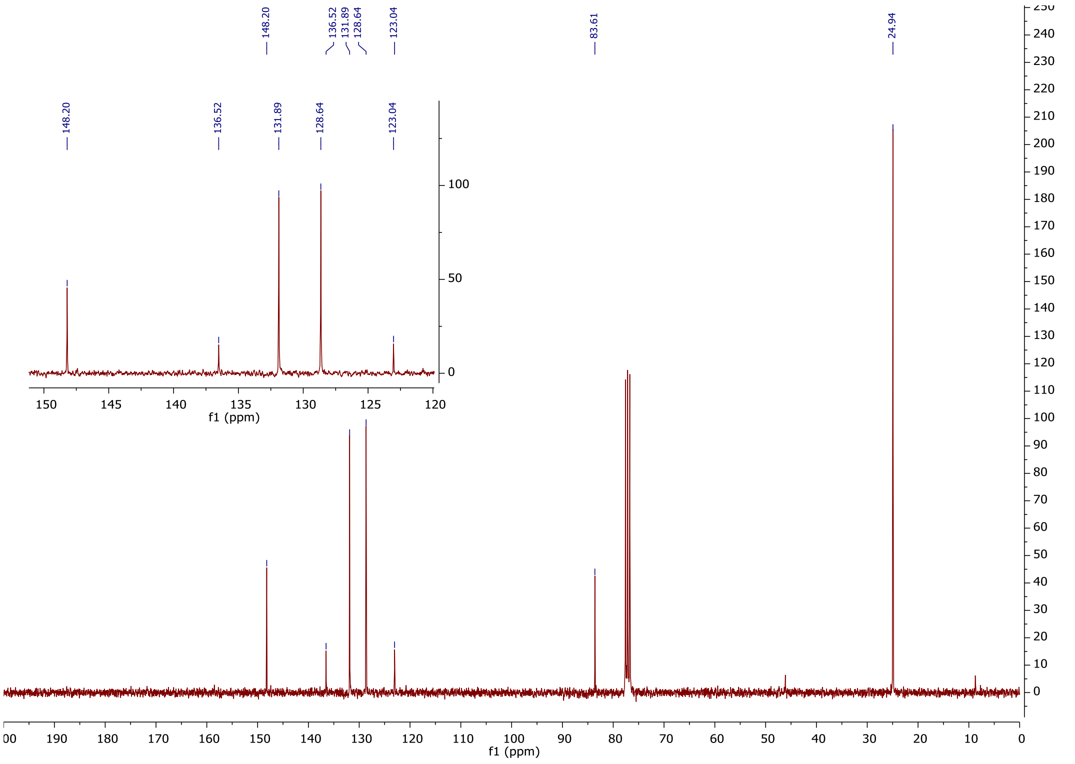

1.4.3. **4-bromonaphthalen-2-yl trifluoromethanesulfonate (300 MHz, $CDCl_3$)**

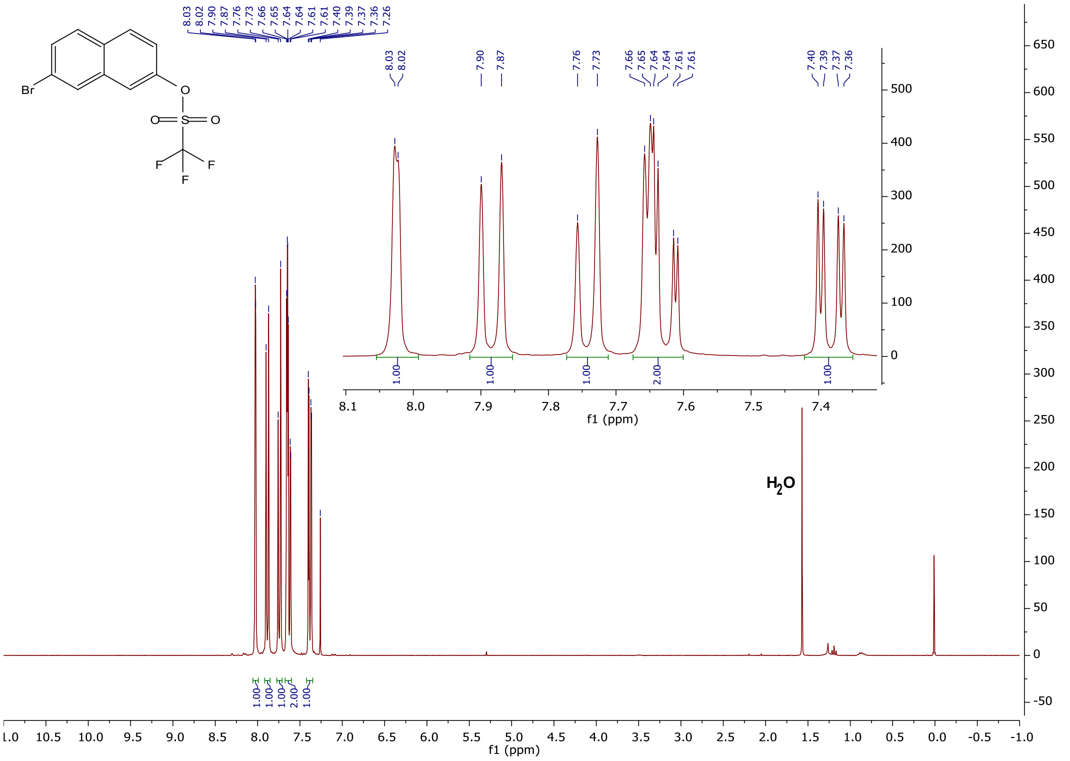

4-bromonaphthalen-2-yl trifluoromethanesulfonate (75 MHz, $CDCl_3$)

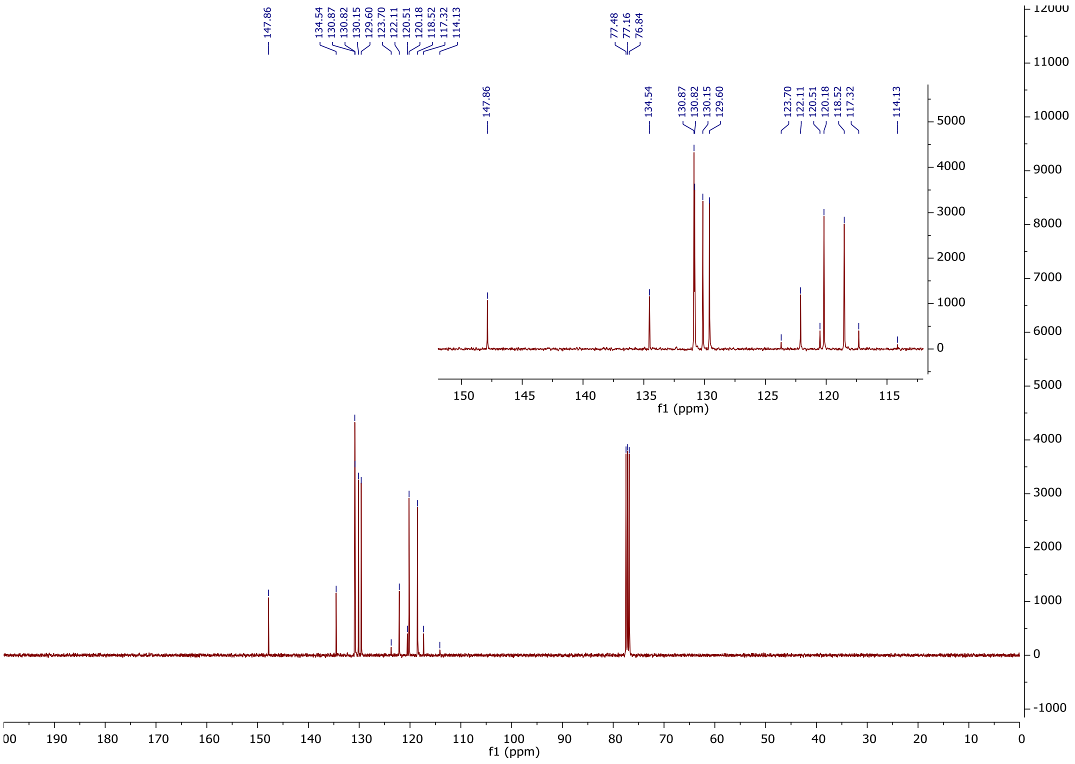

1.4.4. *(E)*-2-bromo-7-(4-bromostyryl)naphthalene (400 MHz, $CDCl_3$)

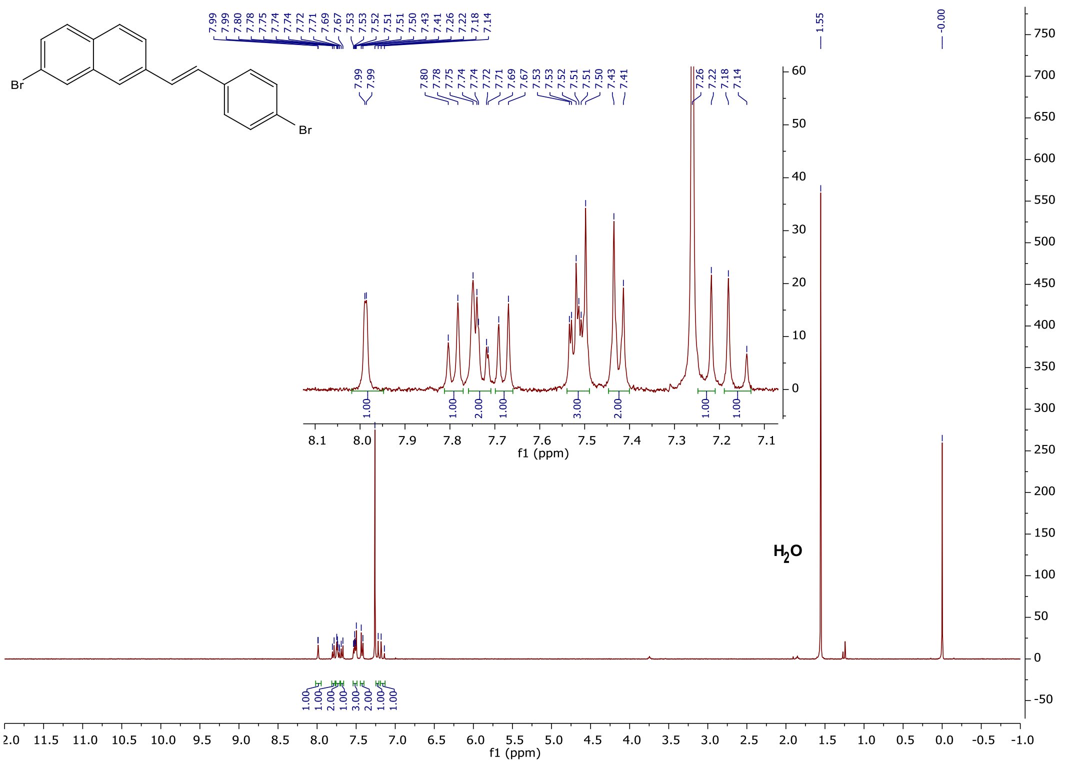

1.4.5. (*E*)-8-(2-(7-bromonaphthalen-2-yl)vinyl)naphtho[1,2-*b*]thiophene (400 MHz, $CDCl_3$)

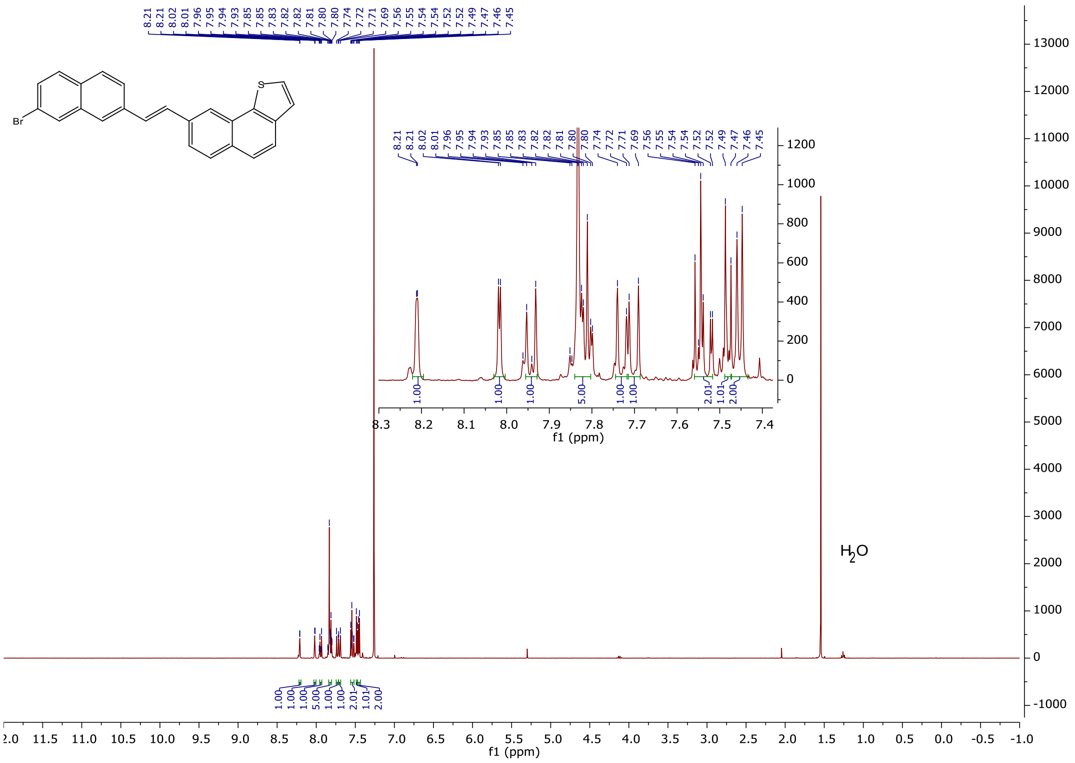

1.4.6. **2,11-dibromobenzo[c]phenanthrene (300 MHz, $CDCl_3$)**

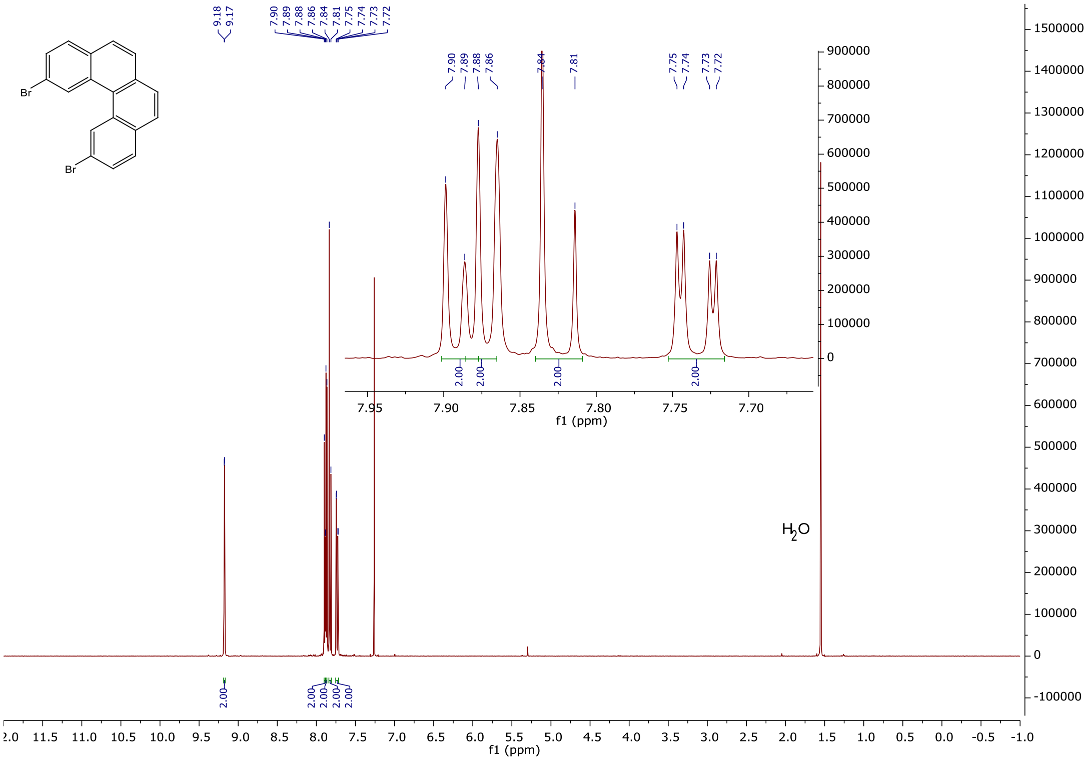

2,11-dibromobenzo[c]phenanthrene (75 MHz, $CDCl_3$)

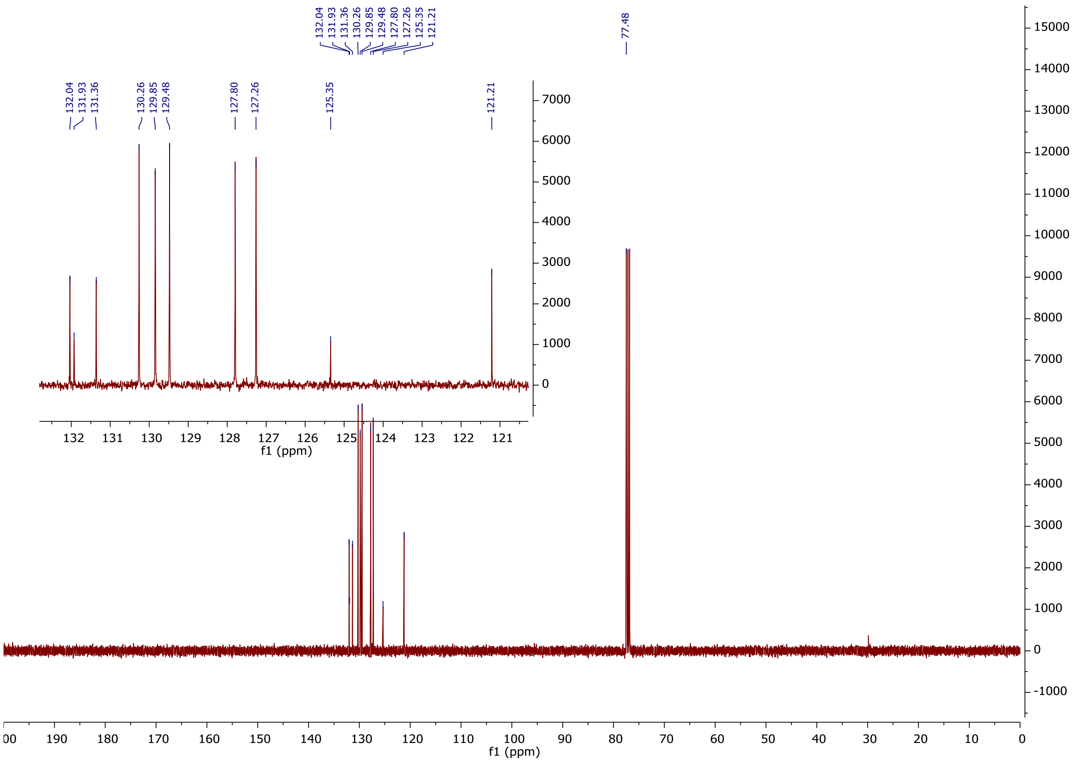

1.4.7. (*E*)-14-(2-(naphtho[2,1-*b*]thiophen-8-yl)vinyl)naphtho[1’,2’:5,6]phenanthro[4,3-*b*]thiophene (400 MHz, $CDCl_3$)

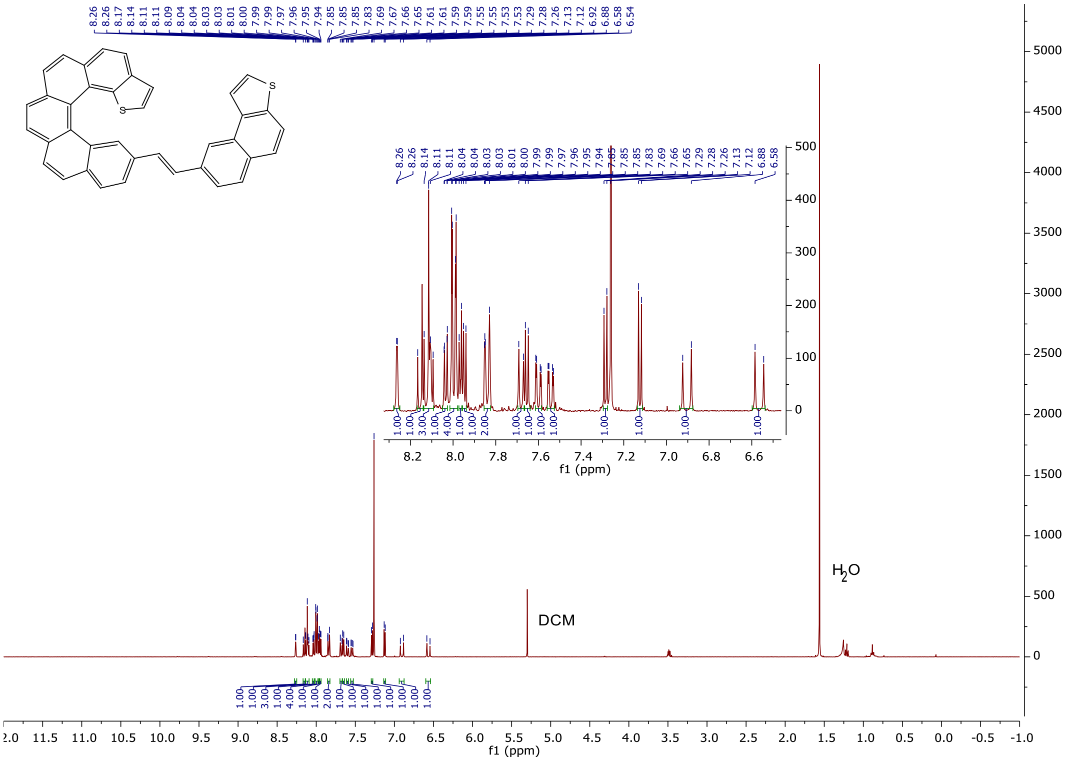

1.4.8. **14-bromonaphtho[1’,2’:5,6]phenanthro[4,3-*b*]thiophene (400 MHz, $CDCl_3$)**

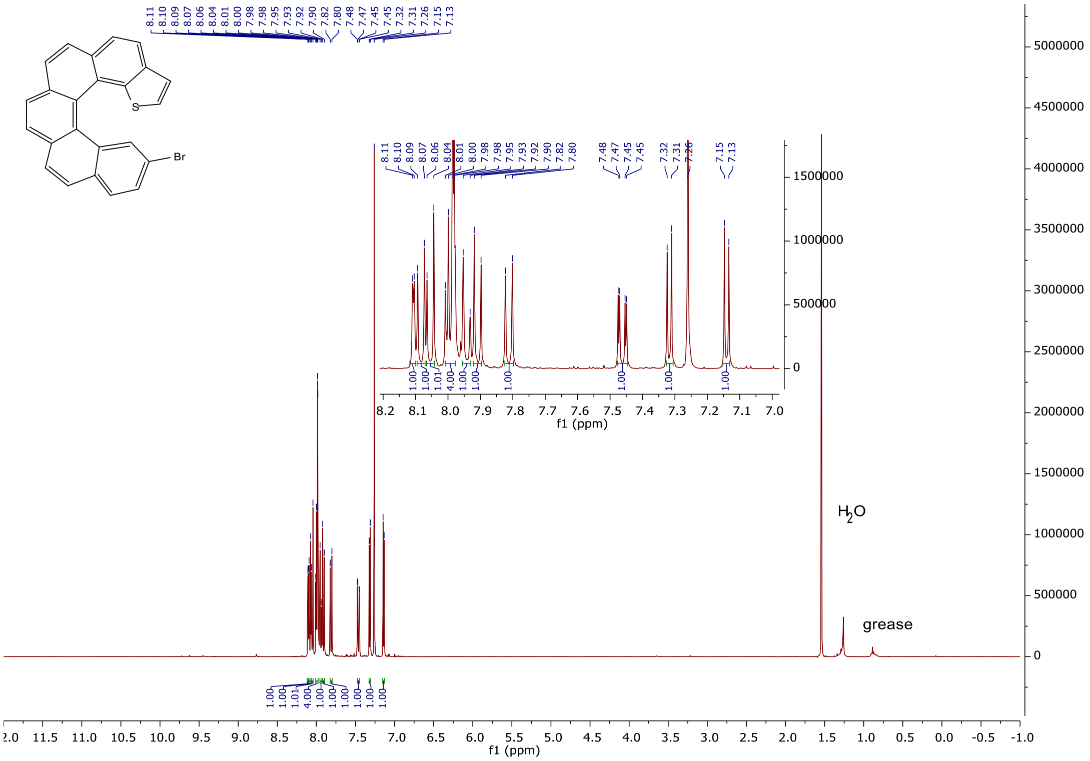

14-bromonaphtho[1’,2’:5,6]phenanthro[4,3-*b*]thiophene (101 MHz, $CDCl_3$)

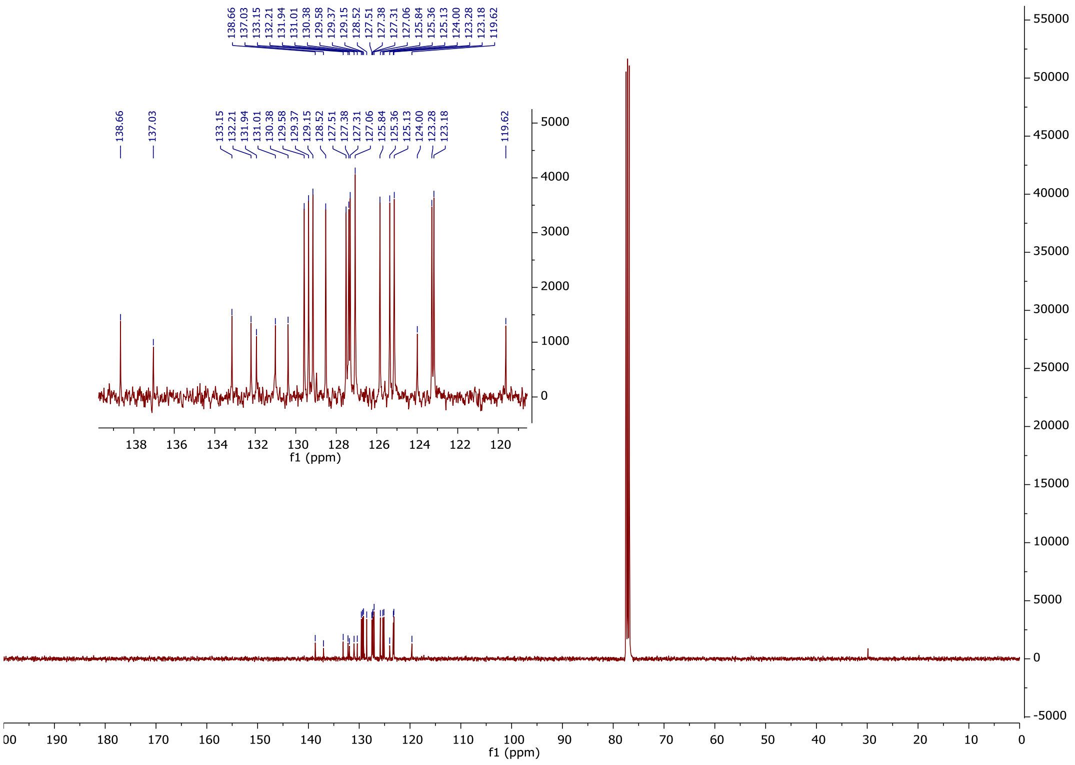

1.4.9. **1,14-dithia[7]helicene or *endo*-*exo*-dithia[7]helicene (400 MHz, $CDCl_3$)**

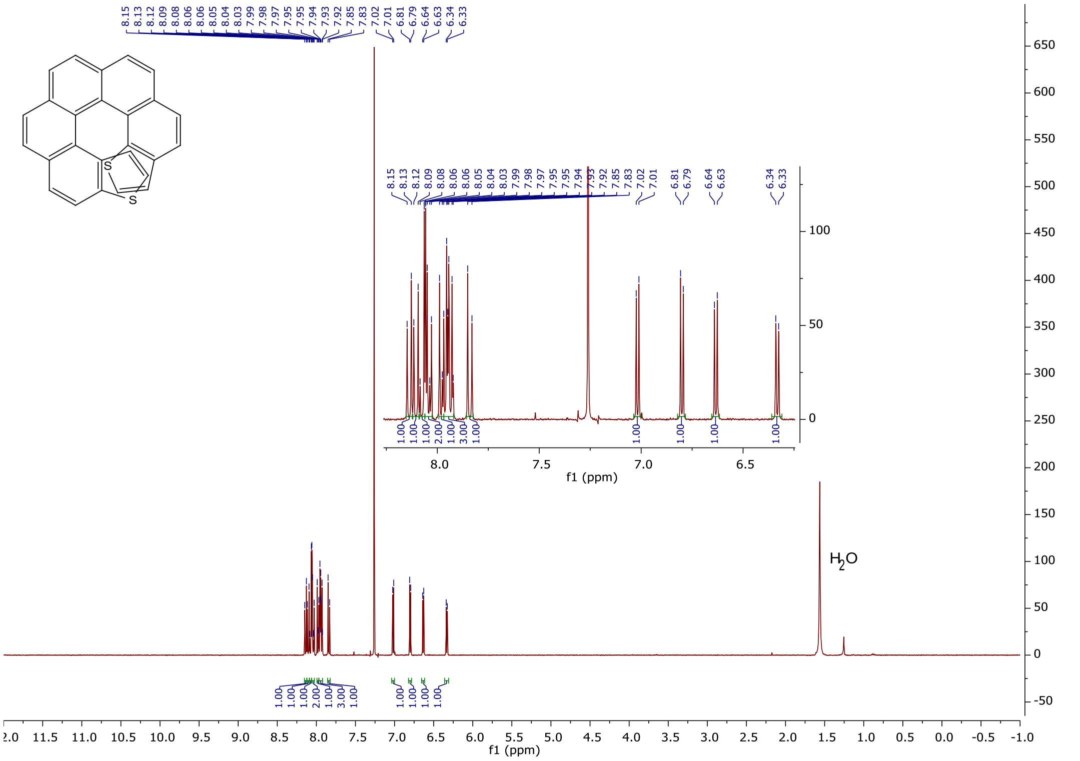

1,14-dithia[7]helicene (101 MHz, $CDCl_3$)

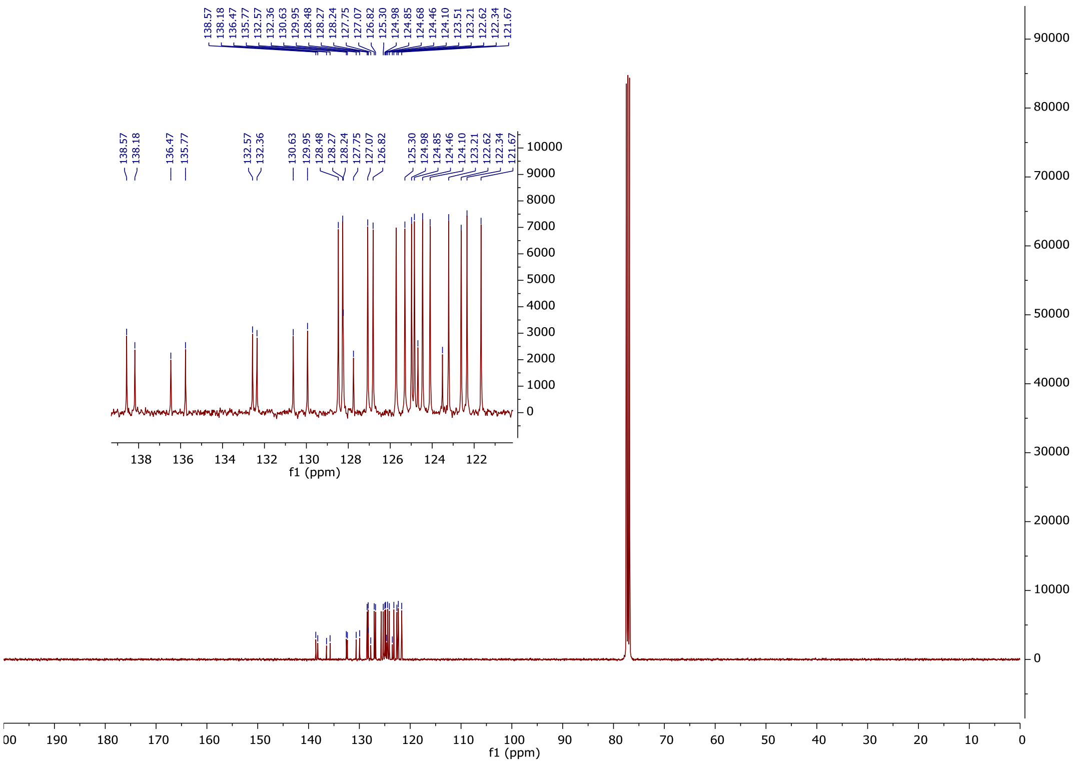

1.4.10. **1,20-dithia[10]helicene or *endo*-*exo*-dithia[10]helicene (400 MHz, $CDCl_3$)**

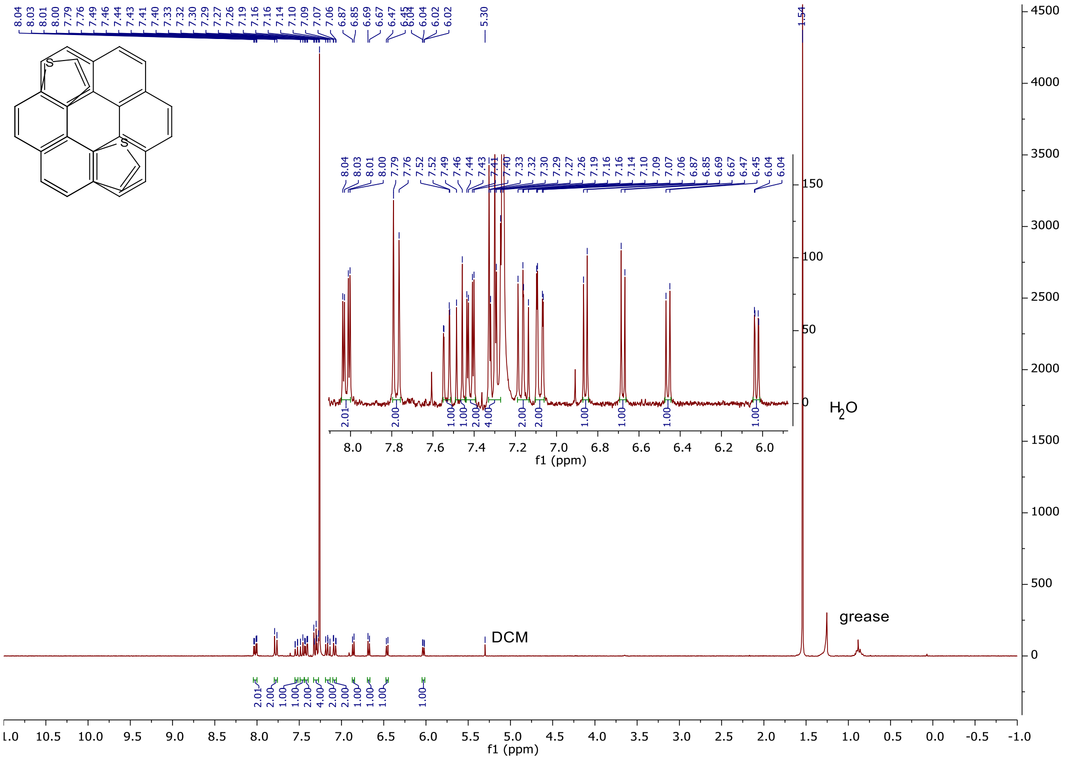

1.4.11. **3,20-Dithia[10]helicene or *exo*-dithia[10]helicene (300 MHz, CDCl$_3$)**

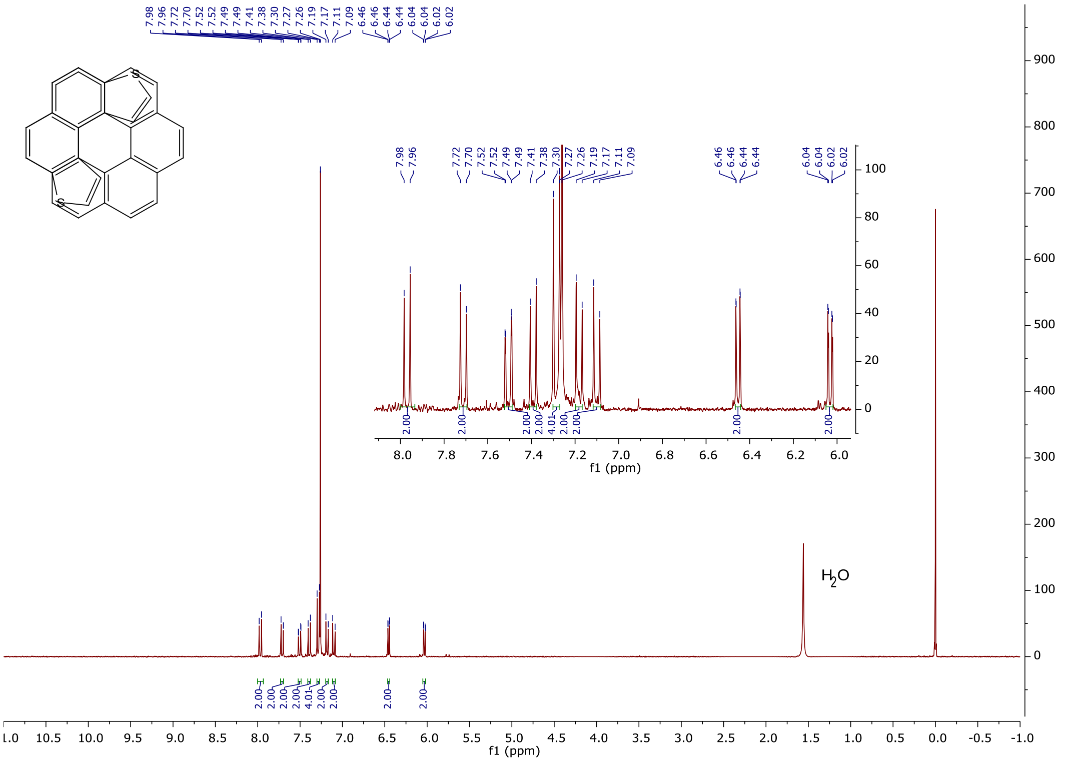

3,20-Dithia[10]helicene or *exo*-dithia[10]helicene (75 MHz, CDCl$_3$)

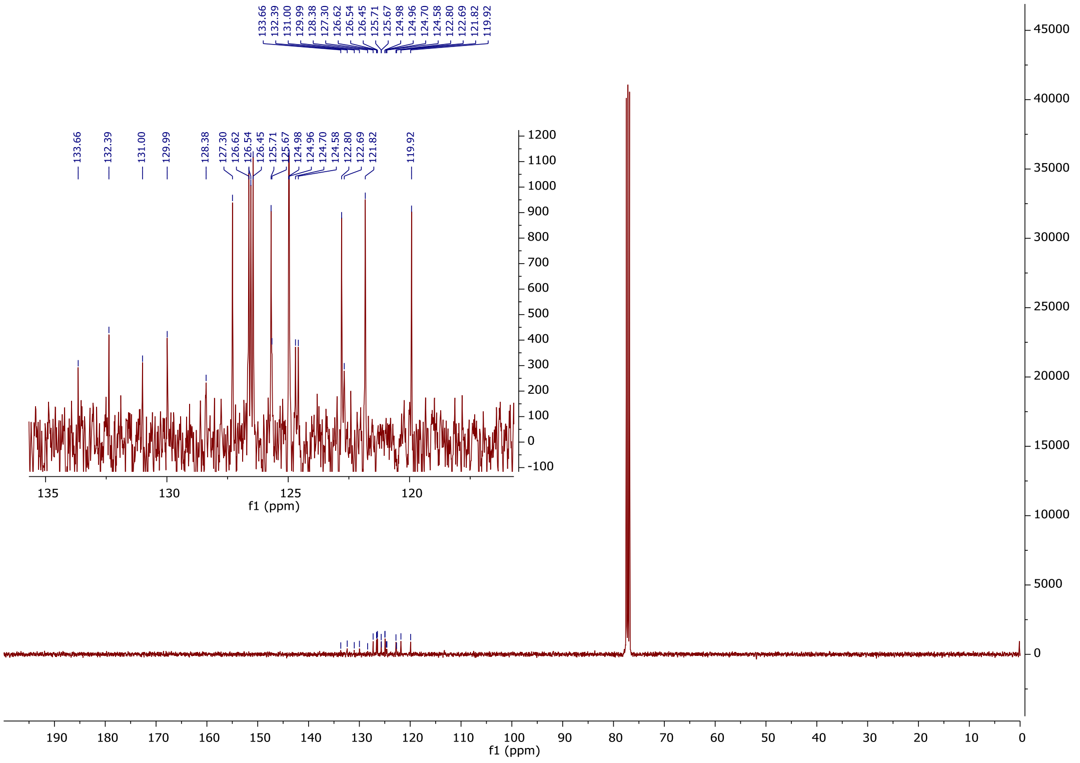

1.4.12. **1,22-Dithia[10]helicene or *endo*-dithia[10]helicene (300 MHz, $CDCl_3$)**

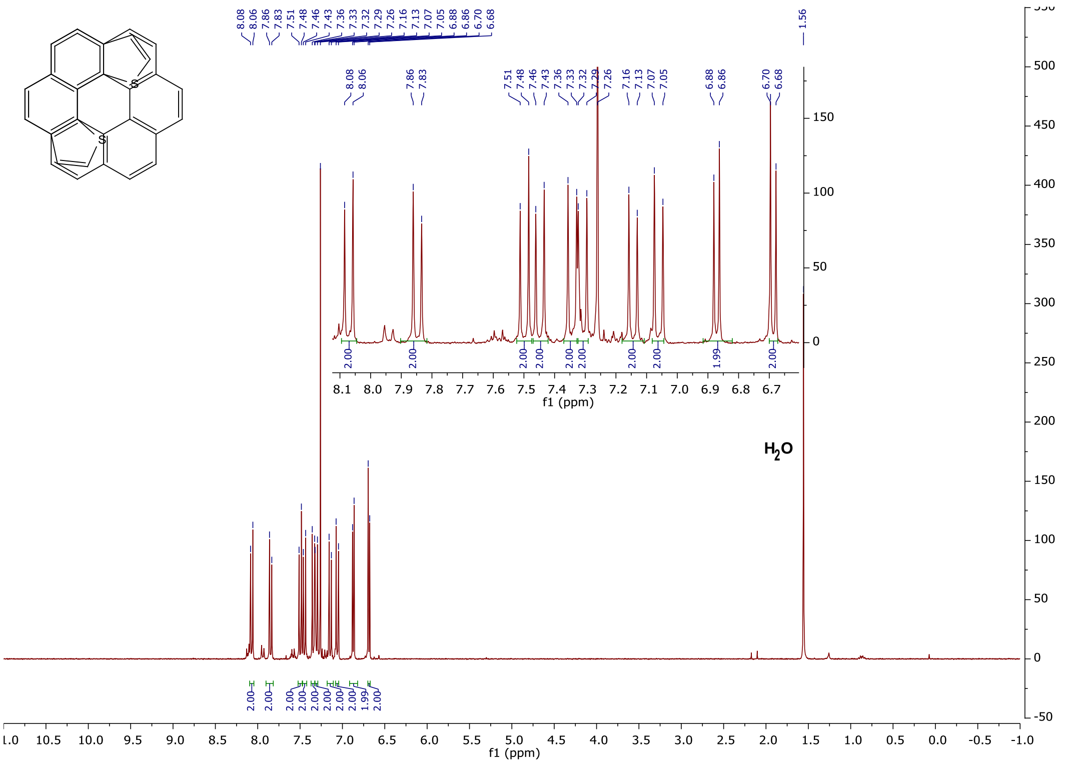

1,22-Dithia[10]helicene or *endo*-dithia[10]helicene (75 MHz, $CDCl_3$)

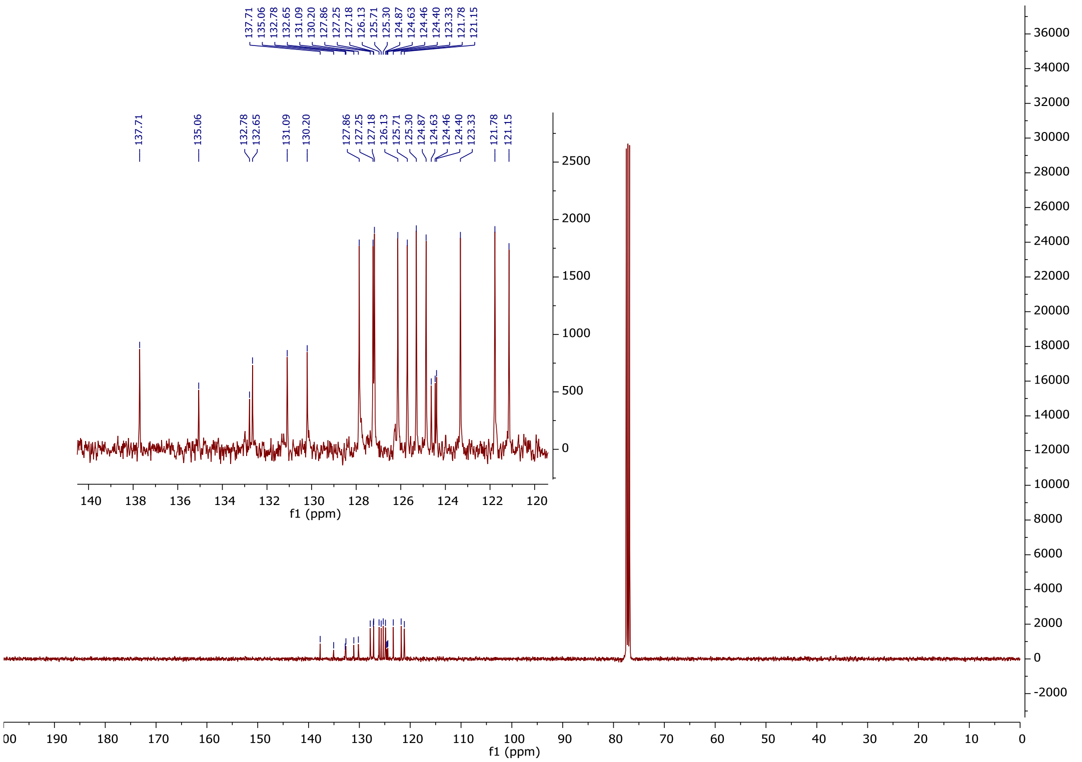

1.4.13. **3,22-Dithia[11]helicene or *exo*-dithia[11]helicene (300 MHz, $CDCl_3$)**

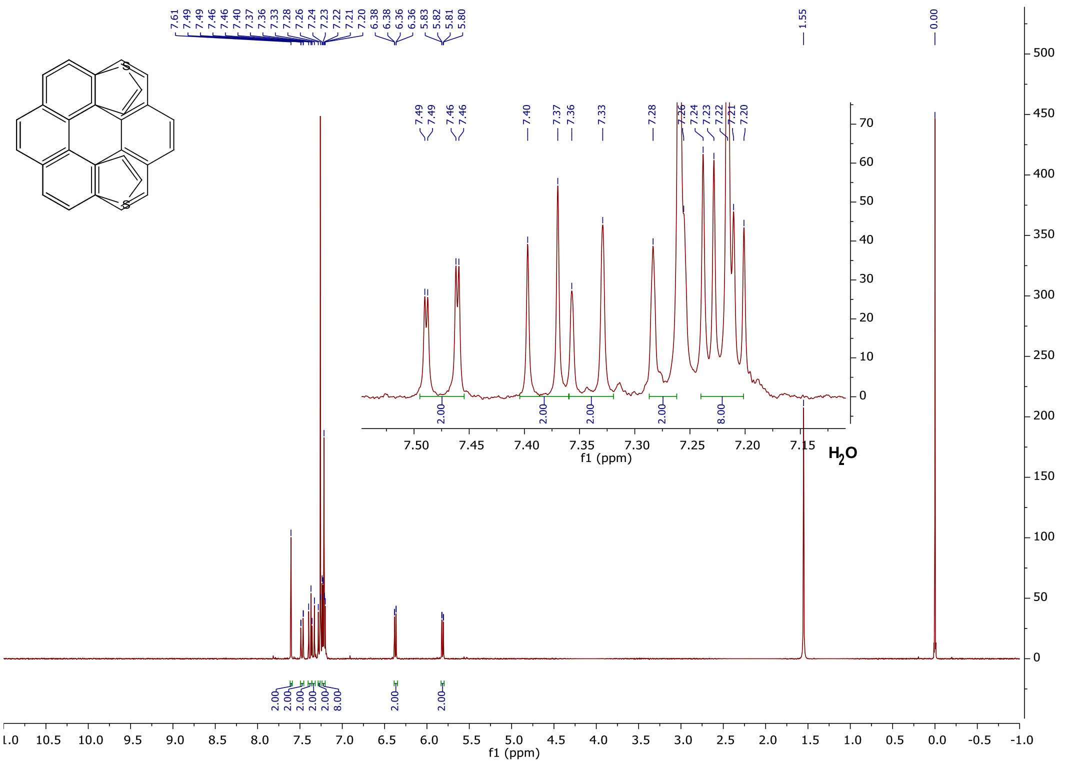

3,22-Dithia[11]helicene or *exo*-dithia[11]helicene (75 MHz, $CDCl_3$)

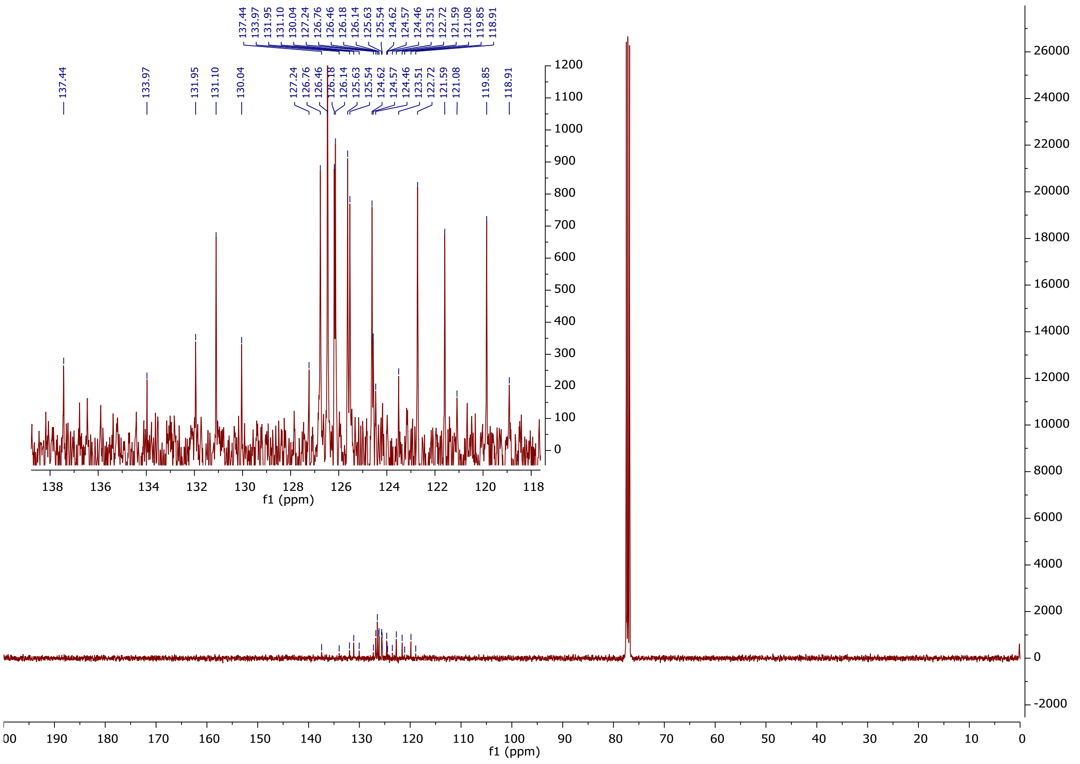

1.4.14. **1,24-Dithia[11]helicene or *endo*-dithia[11]helicene (300 MHz, $CDCl_3$)**

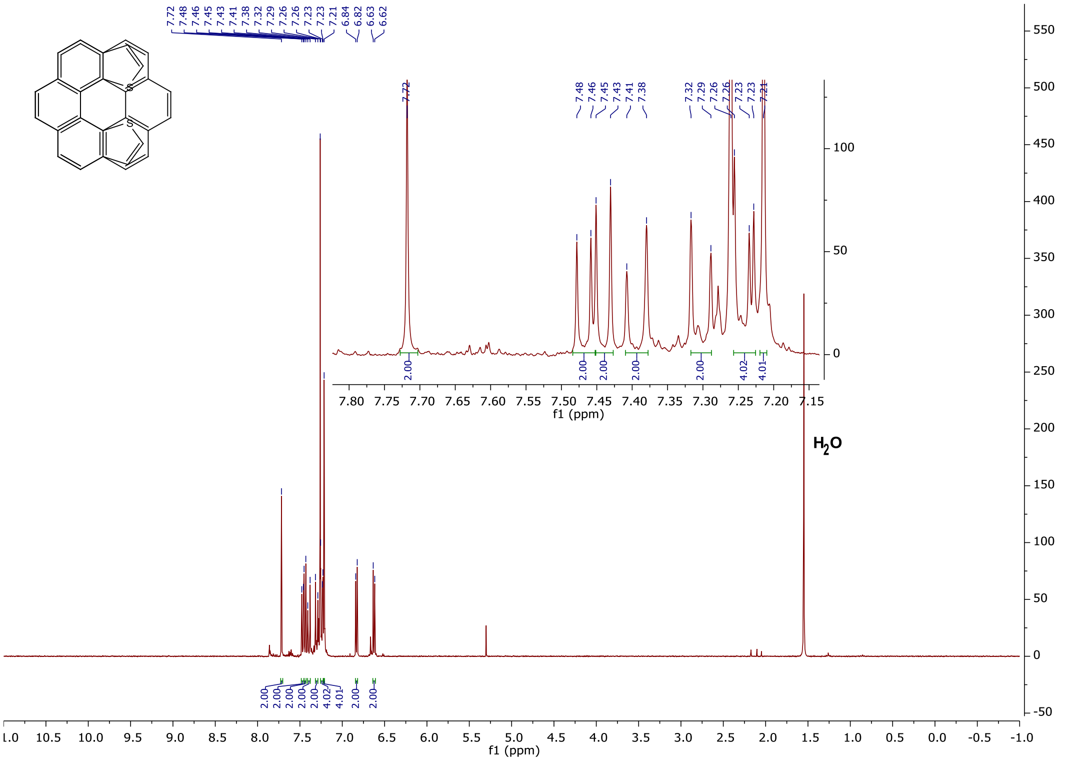

1,24-Dithia[11]helicene or *endo*-dithia[11]helicene (75 MHz, $CDCl_3$)

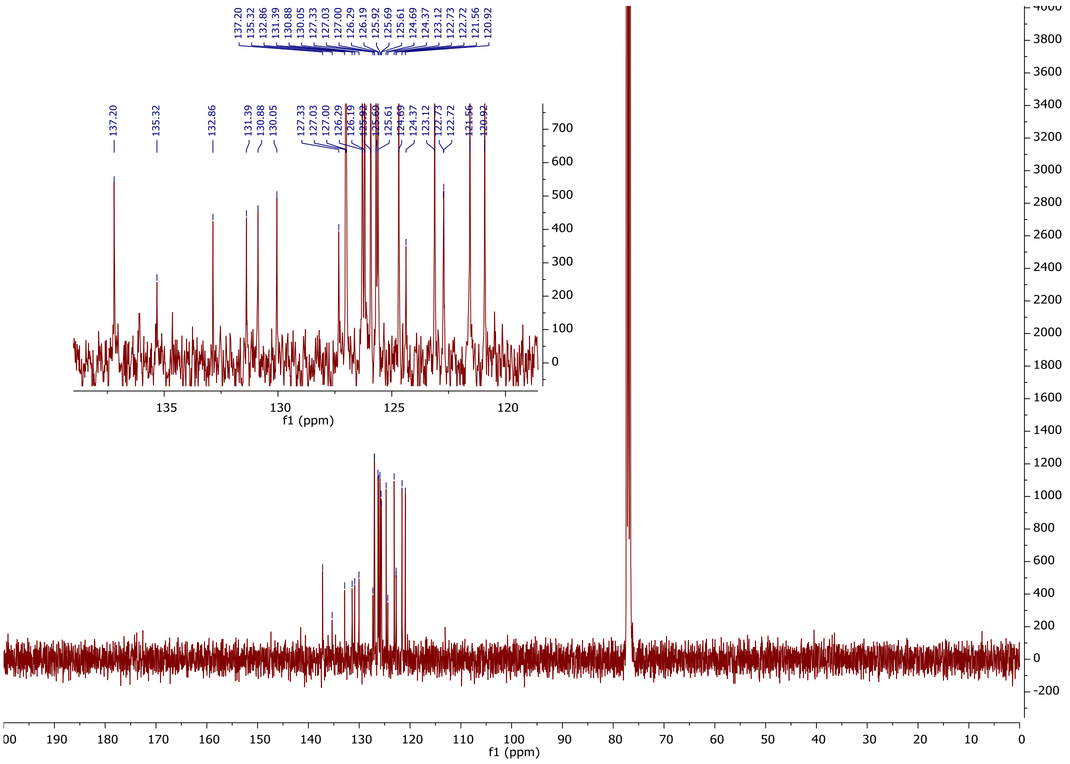

2. Supplementary Figures and Tables

Supplementary Figures 1.

Our group synthesized all the fragments that we needed to synthesize the bis-stilbenic precursors in previous works.[8,9]

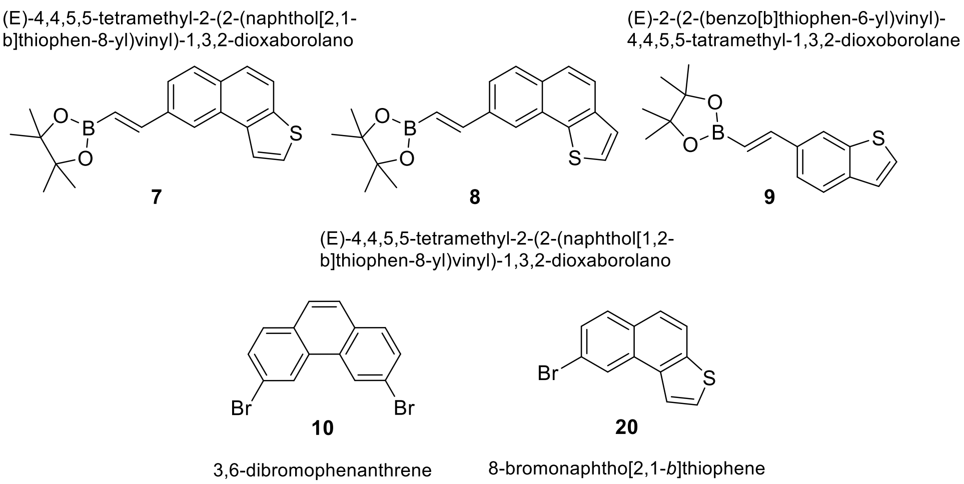

Supplementary Figures 2.

Synthetic pathway of naphthalene-2,7-diyl bis(trifluoromethanesulfonate) **11** and 2,11-dibromobenzo[c]phenanthreno **12**

[8] Baciu, B. C.; de Ara, T.; Sabater, C.; Untiedt, C.; Guijarro, A. *Nanoscale Adv.* **2020**, *2*, 1921.

[9] Baciu. B. C.; Bronk, P. J.; de Ara, T.; Rodriguez, R.; Morgante, P.-P.; Sabater, C.; Autschbach, J.; Crassous, J.; Vanthuyne, N.; Untiedt, C.; Guijarro, A. *J. Mat. Chem. C.* **2022**, 10, 14306.

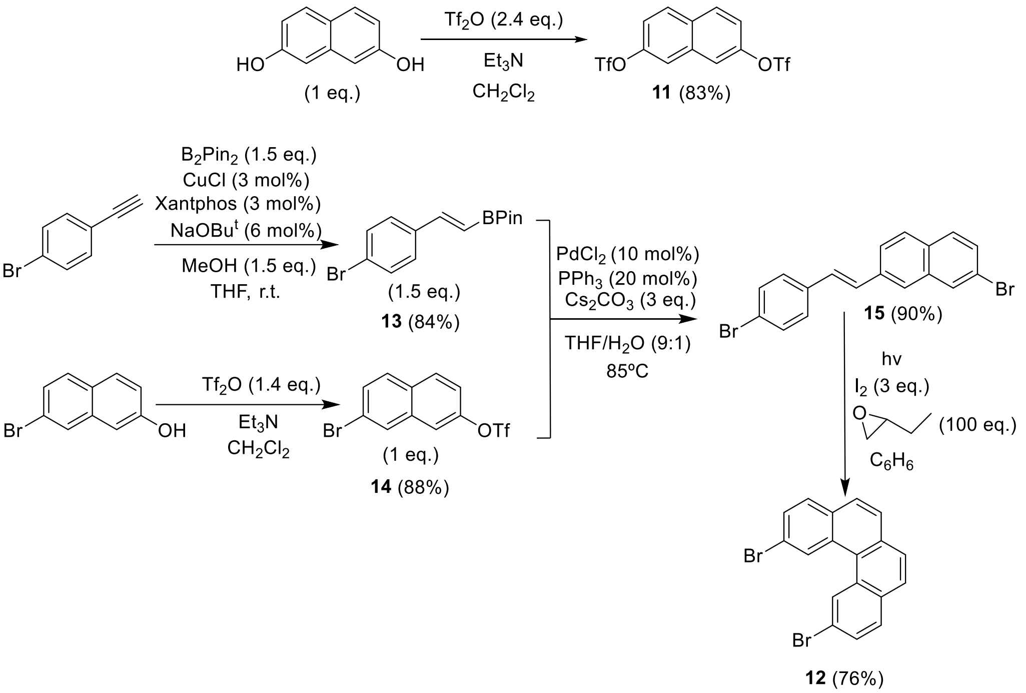

Supplementary Figures 3.

Synthetic pathway of racemic dithia[10]helicenes and dithia[11]helicenes

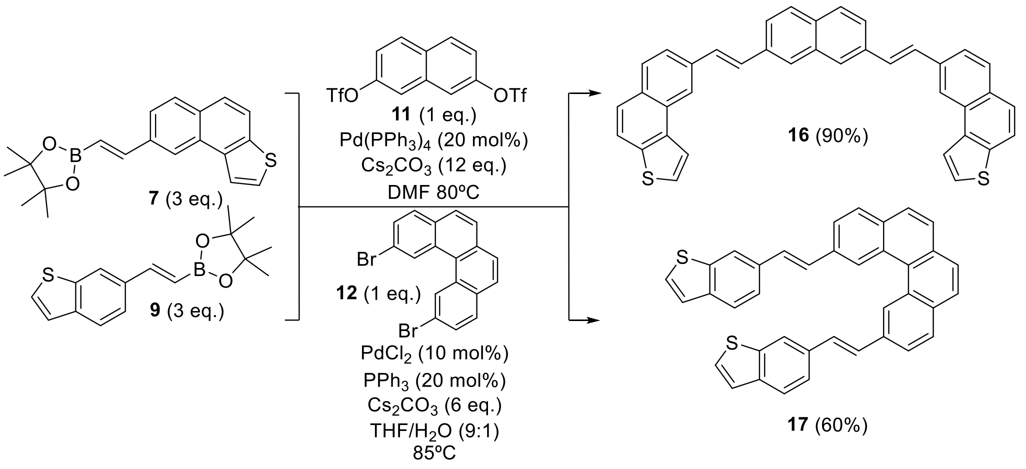

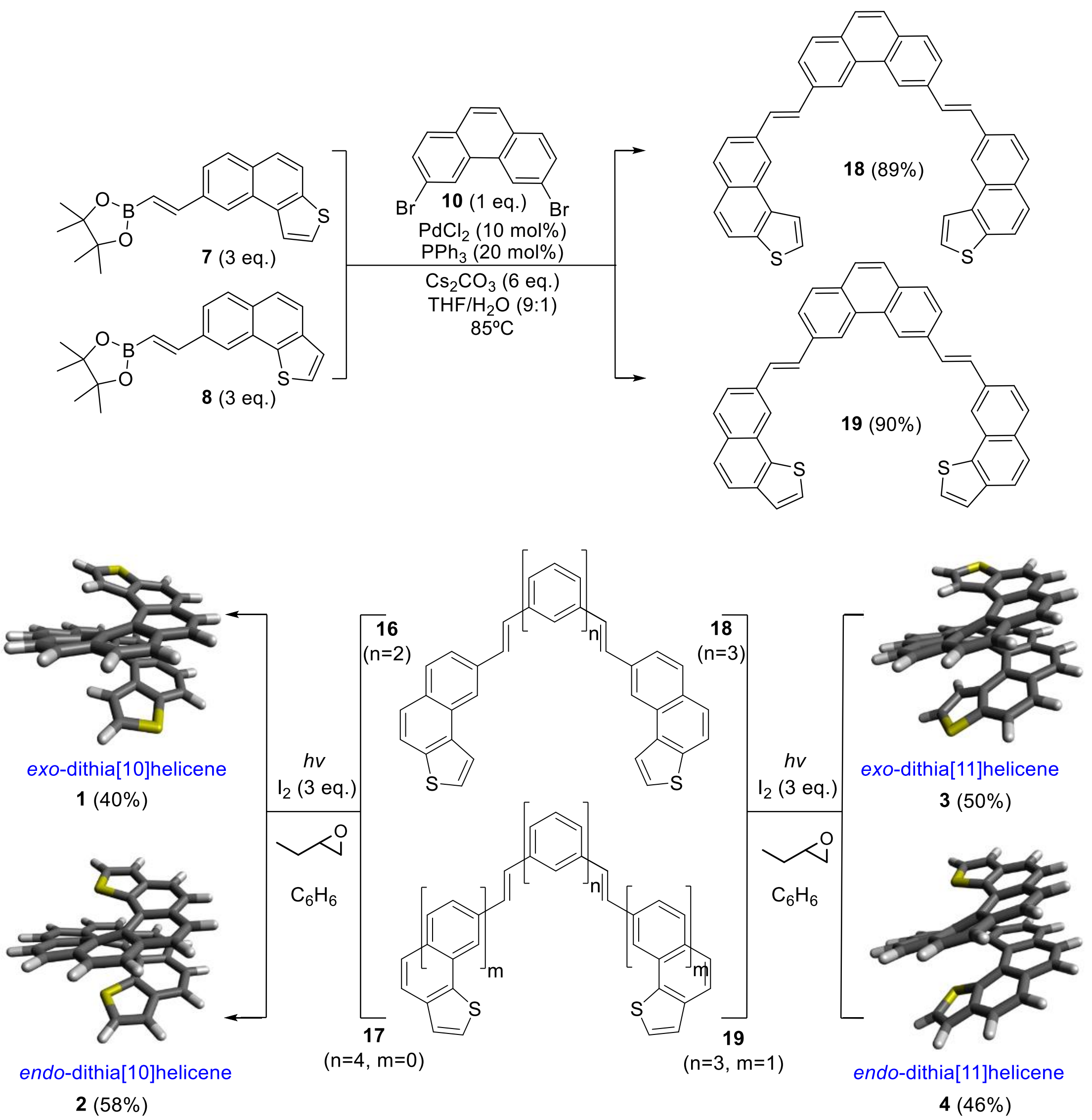

Supporting Figure 4.

Synthetic pathway to obtain *endo*-*exo*-dithia[7] and [10]helicenes

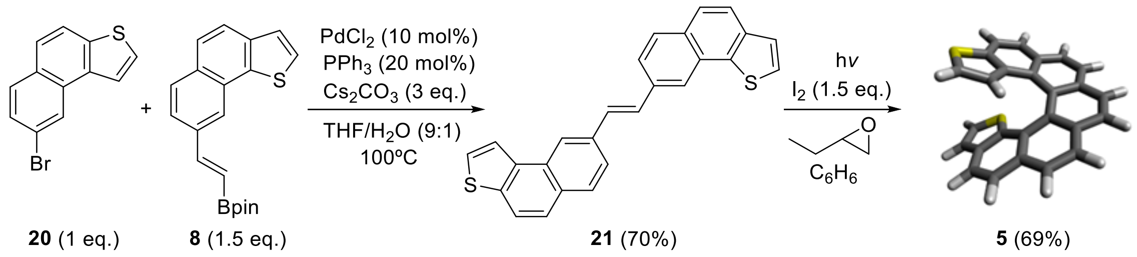

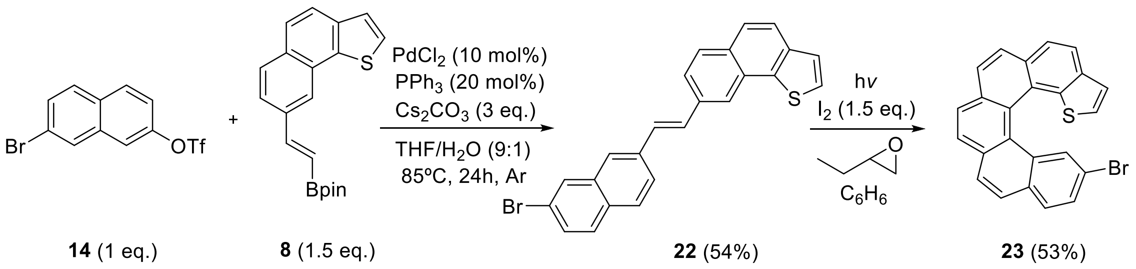
Br
OTf
+
S
Bpin
PdCl2 (10 mol%)
PPh3 (20 mol%)
Cs2CO3 (3 eq.)
THF/H2O (9:1)
85ºC, 24h, Ar
S
Br
hv
I2 (1.5 eq.)
O
C6H6
S
Br
14 (1 eq.)
8 (1.5 eq.)
22 (54%)
23 (53%)

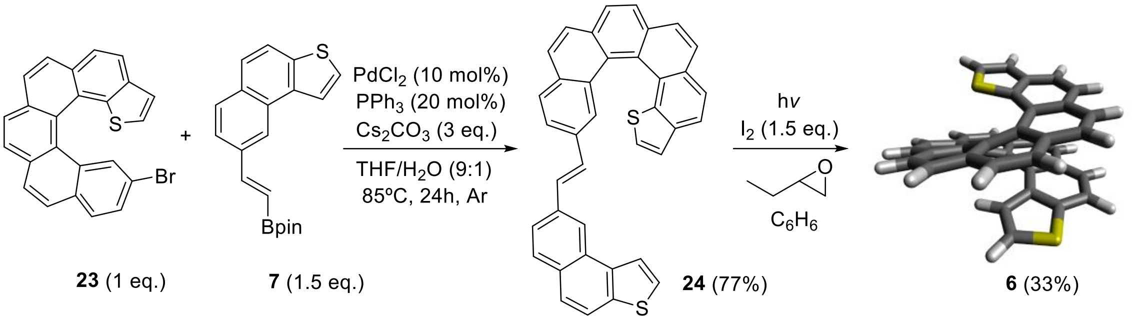
S
Br
+
S
Bpin
PdCl2 (10 mol%)
PPh3 (20 mol%)
Cs2CO3 (3 eq.)
THF/H2O (9:1)
85ºC, 24h, Ar
S
S
hv
I2 (1.5 eq.)
O
C6H6
23 (1 eq.)
7 (1.5 eq.)
24 (77%)
6 (33%)

3. Supporting Table 1. DFT CALCULATIONS OF THE BINDING PROPERTIES TO GOLD

Exo-dithia[10]helicene-Au_{10}

Configuration	*E* (Ha)	$\Delta E_{f\ (uncorrected)}$[a] (kcal/mol)	ΔE_f[b] (kcal/mol)	ΔE_{rel} (kcal/mol)[c]	Notes
S[10]DTH-Au_{10}	-3611.799432	-7.363196473	-7.277855181	0.53	η^1 (S atom)
r1[10]DTH-Au_{10}	-3611.800282	-7.896579548	-7.811238256	0	η^1 (alpha C)
r2[10]DTH-Au_{10}	-3611.797923	-6.416284637	-6.330943345	1.48	η^2
r3[10]DTH-Au_{10}	-3611.798683	-6.893191857	-6.807850565	1.00	η^2
r4[10]DTH-Au_{10}	-3611.798509	-6.784005205	-6.698663912	1.11	η^2
r5[10]DTH-Au_{10}	-3611.798003	-6.466485397	-6.381144105	1.43	η^2
exo-[10]DTH	-2256.873192				C_2 symmetry
Au_{10}	-1354.914506				T_d symmetry

[a] Energy of formation of the complex DTH-Au_{10} (uncorrected). [b] Energy of formation of the complex DTH-Au_{10} (BSSE corrected). [b] Relative energy of formation (BSSE corrected). A basis set superposition error (BSSE) of +0.085341292 kcal/mol was determined in all cases.

Exo-dithia[10]helicene-Au_{10} series

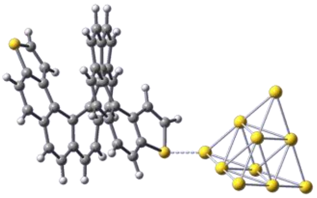	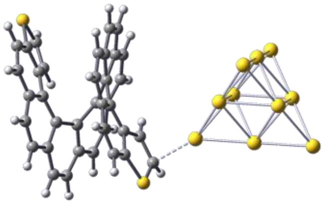	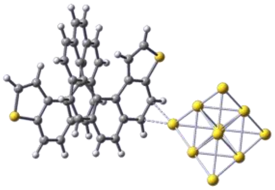
S[10]DTH-Au_{10}	r1[10]DTH-Au_{10}	r2[10]DTH-Au_{10}
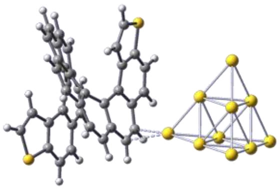	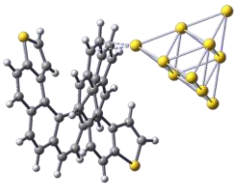	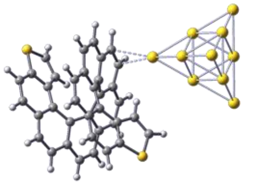
r3[10]DTH-Au_{10}	r4[10]DTH-Au_{10}	r5[10]DTH-Au_{10}

Endo-dithia[10]helicene-Au_{10}

Configuration	*E* (Ha)	$\Delta E_{f\ (uncorrected)}$[a] (kcal/mol)	ΔE_f [b] (kcal/mol)	ΔE_{rel} (kcal/mol)[c]	Notes
S[10]DTH-Au_{10}	-3611.802016	-10.02948434	-9.931592857	-2.15	incidence[d]
r1[10]DTH-Au_{10}	-3611.798585	-7.876499244	-7.778607762	0	η^1 (alpha C)
r2[10]DTH-Au_{10}	-3611.796694	-6.68987878	-6.591987298	1.19	η^2
r3[10]DTH-Au_{10}	-3611.797378	-7.119095278	-7.021203796	0.76	η^2
r4[10]DTH-Au_{10}	-3611.79891	-8.080439832	-7.98254835	-0.20	incidence[d]
r5[10]DTH-Au_{10}	-3611.801057	-9.427702728	-9.329811246	-1.55	incidence[d]
endo-[10]DTH	-2256.871527				C_2 symmetry
Au_{10}	-1354.914506				T_d symmetry

[a] Energy of formation of the complex DTH-Au_{10} (uncorrected). [b] Energy of formation of the complex DTH-Au_{10} (BSSE corrected). [b] Relative energy of formation (BSSE corrected). A basis set superposition error (BSSE) of +0.097891482 kcal/mol was determined in all cases. [d] Molecule lying on several Au atoms resulting in an overestimation of the binding site, rendering it unsuitable for accurate comparisons.

Endo-dithia[10]helicene-Au$_{10}$ series

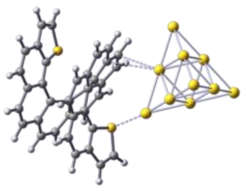	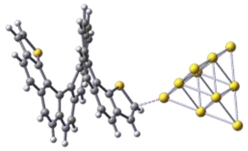	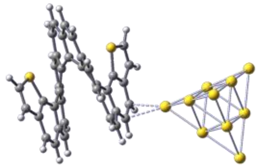
S[10]DTH-Au$_{10}$	r1[10]DTH-Au$_{10}$	r2[10]DTH-Au$_{10}$
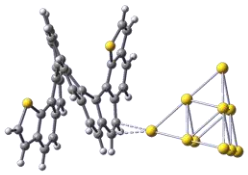	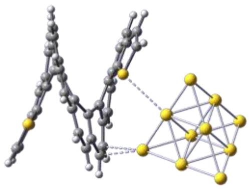	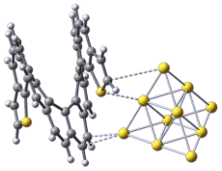
r3[10]DTH-Au$_{10}$	r4[10]DTH-Au$_{10}$	r5[10]DTH-Au$_{10}$

Exo-dithia[11]helicene-Au_{10}

Configuration	*E* (Ha)	$\Delta E_{f\ (uncorrected)}$[a] (kcal/mol)	ΔE_f[b] (kcal/mol)	ΔE_{rel} (kcal/mol)[c]	Notes
S[11]DTH-Au_{10}	-3765.465296	-7.506268639	-7.422182366	0.513302771	η^1 (S atom)
r1[11]DTH-Au_{10}	-3765.466114	-8.01957141	-7.935485137	0	η^1 (alpha C)
r2[11]DTH-Au_{10}	-3765.463714	-6.51354861	-6.429462337	1.5060228	η^2
r3[11]DTH-Au_{10}	-3765.464643	-7.096504935	-7.012418662	0.923066475	η^2
r4[11]DTH-Au_{10}	-3765.464396	-6.941510089	-6.857423816	1.078061321	η^2
r5[11]DTH-Au_{10}	-3765.464423	-6.958452845	-6.874366572	1.061118564	η^2
r6[11]DTH-Au_{10}	-3765.461369	-5.042038832	-4.957952559	2.977532577	
exo-[11]DTH	-2410.538828				C_2 symmetry
Au_{10}	-1354.914506				T_d symmetry

[a] Energy of formation of the complex DTH-Au_{10} (uncorrected). [b] Energy of formation of the complex DTH-Au_{10} (BSSE corrected). [b] Relative energy of formation (BSSE corrected). A basis set superposition error (BSSE) of +0.084086273 kcal/mol was determined in all cases.

Exo-dithia[11]helicene-Au_{10} series

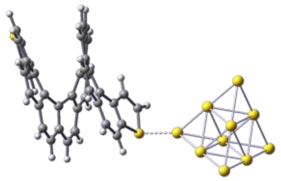	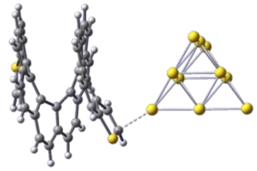	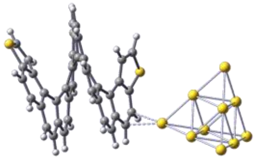
S[11]DTH-Au_{10}	r1[11]DTH-Au_{10}	r2[11]DTH-Au_{10}
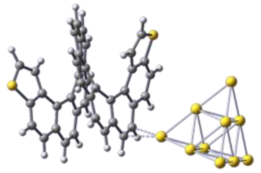	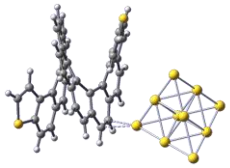	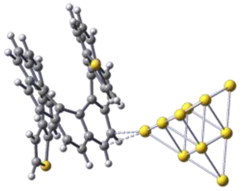
r3[11]DTH-Au_{10}	r4[11]DTH-Au_{10}	r5[11]DTH-Au_{10}
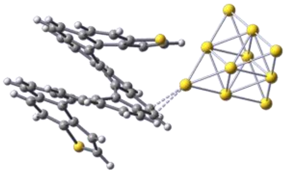		
r6[11]DTH-Au_{10}		

Endo-dithia[11]helicene-Au_{10}

Configuration	E (Ha)	$\Delta E_{f\ (uncorrected)}$[a] (kcal/mol)	ΔE_f[b] (kcal/mol)	ΔE_{rel} (kcal/mol)[c]	Notes
S[11]DTH-Au_{10}	-3765.467365	-10.1587513	-10.07592004	-2.17	incidence[d]
r1[11]DTH-Au_{10}	-3765.463911	-7.991333482	-7.908502228	0	η^1 (alpha C)
r2[11]DTH-Au_{10}	-3765.461978	-6.778357619	-6.695526365	1.21	η^2
r3[11]DTH-Au_{10}	-3765.462849	-7.324918393	-7.242087139	0.67	η^2
r4[11]DTH-Au_{10}	-3765.464306	-8.239199735	-8.156368481	-0.25	incidence[d]
r5[11]DTH-Au_{10}	-3765.466662	-9.717612117	-9.634780863	-1.73	incidence[d]
r6[11]DTH-Au_{10}	-3765.462746	-7.260284915	-7.177453661	0.73	η^2
endo-[11]DTH	-2410.53667				C_2 symmetry
Au_{10}	-1354.914506				T_d symmetry

[a] Energy of formation of the complex DTH-Au_{10} (uncorrected). [b] Energy of formation of the complex DTH-Au_{10} (BSSE corrected). [b] Relative energy of formation (BSSE corrected). A basis set superposition error (BSSE) of +0.082831254 kcal/mol was determined in all cases. [d] Molecule lying on several Au atoms resulting in an overestimation of the binding site, rendering it unsuitable for accurate comparisons.

Endo-dithia[11]helicene-Au_{10} series

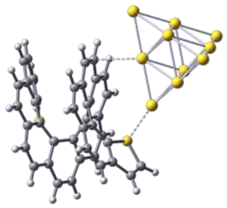	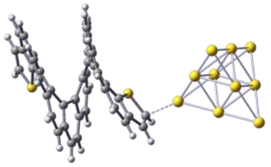	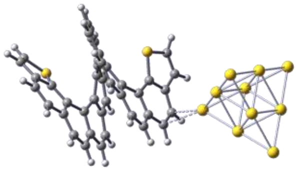
S[11]DTH-Au_{10}	r1[11]DTH-Au_{10}	r2[11]DTH-Au_{10}
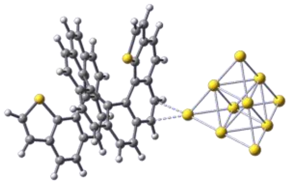	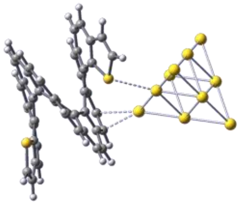	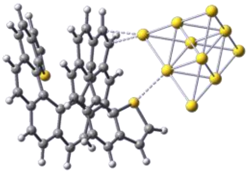
r3[11]DTH-Au_{10}	r4[11]DTH-Au_{10}	r5[11]DTH-Au_{10}
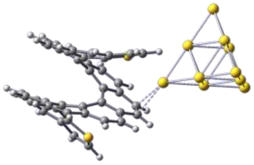		
r6[11]DTH-Au_{10}		

Overall view of ΔE_f (kcal/mol) of *exo*- and *endo*-dithia[10] and [11]helicene-Au$_{10}$ complexes

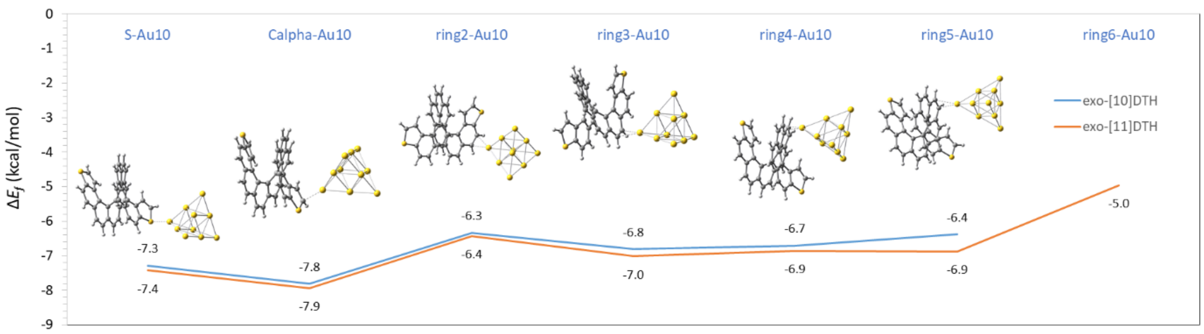

Binding energies of exo-[10]DTH and exo-[11]DTH to a Au10 cluster

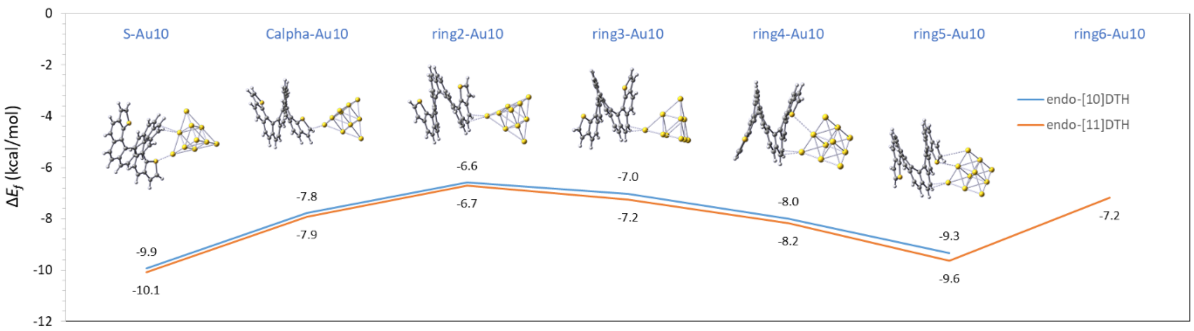

Binding energies of endo-[10]DTH and endo-[11]DTH to a Au10 cluster

Insert figures correspond to the [10]DTH in both graphs

frontiers
